# In silico design and cell-based evaluation of two dual anti breast cancer compounds targeting Bcl-2 and GPER

**DOI:** 10.1038/s41598-023-43860-x

**Published:** 2023-10-20

**Authors:** Loreley-A. Morelos-Garnica, Sonia Guzmán-Velázquez, Itzia-I. Padilla-Martínez, José-R. García-Sánchez, Martiniano Bello, Norbert Bakalara, David Méndez-Luna, José Correa-Basurto

**Affiliations:** 1 Laboratorio de Diseño y Desarrollo de Nuevos Fármacos e Innovación Biotecnológica, Escuela Superior de Medicina, Instituto Politécnico Nacional, Plan de San Luis y Díaz Mirón s/n, Col. Casco de Santo Tomas, Alcaldía Miguel Hidalgo, C.P. 11340 Mexico City, México; 2 Departamento de Fisiología, Escuela Nacional de Ciencias Biológicas, Instituto Politécnico Nacional, Zacatenco, Av. Wilfrido Massieu 399, Col. Nueva Industrial Vallejo, Alcaldía Gustavo A. Madero, C.P. 07738 Mexico City, México; 3 Laboratorio de Química Supramolecular y Nanociencias, Unidad Profesional Interdisciplinaria de Biotecnología, Instituto Politécnico Nacional, Av. Acueducto s/n, Barrio la Laguna Ticomán, Alcaldía Gustavo A. Madero, C.P. 07340 Mexico City, México; 4 Laboratorio de Oncología Molecular y Estrés Oxidativo, Escuela Superior de Medicina, Instituto Politécnico Nacional, Plan de San Luis y Díaz Mirón, s/n, Col. Casco de Santo Tomas, Alcaldía Miguel Hidalgo, C.P. 11340 Mexico City, México; 5 Centre National de la Recherche Scientifique, École Nationale Supérieure de Technologie des Biomolécules de Bordeaux INP, Univeristé de Bordeaux, 146 Rue Léo Saignat, 33000 Bordeaux, France

**Keywords:** Computational chemistry, Drug discovery, Medicinal chemistry, Cancer, Breast cancer

## Abstract

According to WHO statistics, breast cancer (BC) disease represents about 2.3 million diagnosed and 685,000 deaths globally. Regarding histological classification of BC, the Estrogen (ER) and Progesterone (PR) receptors negative-expression cancer, named Triple-Negative BC (TNBC), represents the most aggressive type of this disease, making it a challenge for drug discovery. In this context, our research group, applying a well-established Virtual Screening (VS) protocol, in addition to docking and molecular dynamics simulations studies, yielded two ligands identified as **6** and **37** which were chemically synthesized and evaluated on MCF-7 and MDA-MB-231 cancer cell lines. Strikingly, **37** assayed on MDA-MB-231 (a TNBC cell model) depicted an outstanding value of 18.66 μM much lower than 65.67 μM yielded by Gossypol Bcl-2 inhibitor whose main disadvantage is to produce multiple toxic effects. Highlighted above, enforce the premise of the computational tools to find new therapeutic options against the most aggressive forms of breast cancer, as the results herein showed.

## Introduction

Deregulated apoptosis is a feature now considered as a hallmark of cancer cells. It results from an adaptation to high levels of oncogenic signaling by two main processes: disabling their senescence and de-regulating apoptosis-inducing circuitry^1,2^. Rewired apoptosis machinery considers modification of the Bcl-2 regulatory proteins. Bcl-2 protein (archetype of the family) and other members as Bcl-x_L_, Bcl-w, and Mcl-1 A1, inhibit apoptosis by binding (in part) to two proapoptotic triggering proteins (Bax and Bak)^3,4^. Certainly, death or cell survival depends largely on the ratio of Bcl-2 family anti- and pro-apoptotic proteins. As occurs in most tumor and cancer cell lines, there is an overexpression of the anti-apoptotic Bcl-2 proteins, for example, it is well-known that prostate, colorectal and breast cancers overexpressed Bcl-2 proteins^5–7^. Besides, although there are potent inhibitors of the Bcl-2 family proteins as ABT-737, its molecular structure contributes to its poor drug-profile related to stability, affinity, and metabolism, which led to its failure in preclinical studies^8,9^. In the same way, Gossypol, a polyphenol derived from the cottonseed plant, has demonstrated behavior as pan–Bcl-2 family inhibitor, which, in combination with ABT-737 has overcoming resistance assayed on a clinically relevant leukemia model evaluating a Bcl-2 inhibitor alone^10^. Recently, a new estrogen membrane-receptor named GPER has been described in breast cancer cells, which promotes both cancer cell viability and Tamoxifen-resistance^11,12^. Several studies have strengthened the premise of the pro-oncogenic role of GPER. Firstly, it is required the GPER-activation for survival of xenograft-derived cancer stem cells and progression to metastatic disease, secondly, GPER leads to EGFR spread, transactivation, and responsiveness to growth factors as a result of their signaling, finally, patient outcome and treatment-response of breast cancer-affected patients are most hopeful in GPER negative cancers^13–17^. Since cancer continues to represent a major problem of public health due in part to the fact that single target agents do not affect the complex biochemical processes e.g., back-up or feed-back mechanism from the pathology, or for the drug cocktails given to the patients, which leads to a non-adherence to the treatment, multi-target approaches arise as a hopeful option to find new therapeutic strategies^18,19^. The above arises in part due to the lack of targeted therapies and the poor prognosis of patients with TNBC, which is characterized by high heterogeneity, high rates of metastasis and by representing about 15–24% of diagnosed breast cancers. The immunohistochemical profiling of TNBC is referred to as a lack of expression of estrogen receptors (ER), progesterone receptors (PR), and human epidermal growth factor receptor 2 (HER2)^20^. Therefore, the challenge to face off TNBC lies in developing targeted therapies aimed at simultaneous blockade of oncogenic drivers that promote cell proliferation and survival, avoiding several disadvantages of the current therapy such as surgery, radiation therapy, chemotherapy and polypharmacology with its multiple side effects^21,22^. In this context, bioinformatic tools could screen potential molecules as ligands to reach promising pharmacological targets involved in several pathologies. Hence, from an atomistic perspective, analysis of several protein motions by molecular dynamics simulations yield insights about the conformational rearrangements that a protein suffers in a studied frame time, such information then could be employed to conduct molecular docking studies in which a wide range of ligands (from databases or designed de novo) can be analyzed to select, based on parameters as binding mode, binding energy, metabolism prediction, physicochemical and Absorption/Distribution/Metabolism/Excretion (ADME)-Toxicological data analysis, the most promising ligands to assay on in vitro or in vivo models^23–25^. Hence, in our research group employing a well-established protocol of virtual screening, in addition to combining docking and molecular dynamics simulations, were designed, synthesized, and assayed in vitro, two new compounds herein identified as **6** and **37**, aimed to block the deregulated activity of GPER and Bcl-2 on two cell lines of breast cancer. Thus, in silico simulations depicts the ability of both compounds to reach the binding sites onto the two pharmacological targets with favorable energetic values interacting with the pivotal residues described for GPER and Bcl-2 as is the phenylalanine cluster (Phe206, Phe208, Phe223, and Phe278) and the BH3-BH1 domains, respectively. Whereas, for the antiproliferative activity analysis, the MDA-MB-231 (a cell model of a Triple-Negative Breast Cancer (TNBC)) was the most sensitive cell than MCF-7, correlating with an overexpression of GPER and Bcl-2 blocking activity for the first one. Noteworthy, these findings place **6** and **37** as potential therapeutic options against most aggressive breast cancer cell types as it is TNBC.

## Material and methods

### Rational design of new dual GPER/Bcl-2 ligands

On the chemical basis of a GPER pharmacophore composed of a tetrahydroquinoline scaffold, we previously developed a GPER ligand named G1-PABA^26–28^ which contains in its structure a carboxylic acid group capable of being modified by an amide bond formation. Taking advantage of that, we proposed functionalizing it by forming an amide bond with nucleophilic amines, which contain chemical moieties in its structure, capable of reaching the Bcl-2 binding site^29,30^ and, consequently, blocks both GPER and Bcl-2 aberrant activity.

### Virtual screening protocol

Following a well-established protocol developed by our work group, we based our virtual screening (VS) procedure on the analysis of several features ranging from protein structural parameters as binding modes and energetics yielded by molecular docking, and physicochemical, toxic-biological, and metabolism-prediction by combining various online servers, to select the most promising ligands for their subsequent obtaining by chemical synthesis. In detail, the most relevant values for the VS protocol were the yielded by docking simulations; in this sense, we set for that the binding energy value (in absolute value = $\left|BE\right|$) and percentage of similarity in the binding mode value ($\%similarityBM$), which is the result of the comparison of the tested ligands versus compounds with a demonstrable affinity towards our protein targets, for such control compounds were G1-PABA for GPER and Gossypol for Bcl-2, such analysis considered the results yielded from the following equation:

$$\begin{aligned} & \% ~similarity BM~ = interacting~residues~of~control~compounds - 100 \\ & interacting~residues~of~tested~ligands - X \end{aligned}$$

Results of both parameters are defined as $\left| {BE} \right|$ and $\%similarityBM$ were summative to the final score for the selection of the best ranked ligands. Besides, following the VS protocol, we considered the physicochemical properties analyzed with Molinspiration server https://www.molinspiration.com/, considering for that, the parameters encompassed into Lipinsky rules. Accordingly, for the final score we considered the values yielded by the tested ligands according to: molecular weight 200–500 g/mol, ligands with values out this range would add 0 points, meanwhile, ligands with values into this range would add 10 points; Log P 2–5, ligands with values out this range would add 0 points, meanwhile, ligands with values into this range would add 10 points; H bond acceptors 5–10, ligands with values out this range would add 0 points, meanwhile, ligands with values into this range would add 10 points; H bond donors 2–5, ligands with values out this range would add 0 points, meanwhile, ligands with values into this range would add 10 points. Regarding to toxic-biological properties, these were examined with Osiris Property explorer https://www.organic-chemistry.org/prog/peo/ and considered for the VS protocol setting for all the tested parameters (mutagenicity, teratogenicity, tumorigenicity and irritability) the follow scoring: ligands with high risk would add 0 points, intermedium risk would add 10 points and low risk would add 20 points. Ultimately, all the concerning to predictive properties of the first step metabolism (mediated by the super metabolizing family enzymes CYP450) was conducted with Metaprint2D included in Bioeclipse 2.6.2 (https://www.bioclipse.net/metaprint2d) for the final score of the VS protocol, the possible formation of toxic metabolites according to their biotransformation, hence, for all the tested ligands the scoring parameters were: high predisposition to toxic-metabolite formation would subtract 20 points, medium predisposition to toxic-metabolite formation would subtract 15 points and low predisposition to toxic-metabolite formation would subtract 10 points. Data resulting from the VS protocol is collected in Table S1 on Supplementary Information.

### Ligand construction and molecular docking simulations

Ligand drawing was carried out employing ACD/ChemSketch (Freeware) 2020.1.2^31^ generating in this software a previous 3D pre-optimization to each ligand .mol file, conversion of the .mol file to a Z-matrix was conducted with GaussView 5.0^32^ setting an AM1 semiempirical method to develop a complete energy and geometrical minimization, and a correct proton assignation to each atom that requires it, employing the Gaussian 09^33^ quantic-chemical package, the resulting output files were employed as .pdb input files to molecular docking simulations. Molecular docking simulations were conducted with AutoDock 4.2.6^34^ software employing GPER and Bcl-2 3D models previous generated and validated by our research group and published elsewhere^26–30^, setting the follow parameters prior to docking simulations: polar hydrogens were adequately placed over the atoms capable of establishing polar contacts in the ligand/receptor complexes, and Kollman charges were assigned to the whole proteins. For the ligands, Gasteiger charges were assigned, and the affinity maps were generated using a grid-based procedure delimiting a grid box covering most of the protein with dimensions of 126 Å^3^ in each XYZ coordinates (blind docking procedure) with a grid spacing of 0.375 Å^3^. As scoring sampling was selected the Lamarckian genetic algorithm including a randomized initial population of 100 individuals delimiting a unique as top individual that survived, and a maximum number of energy evaluations of 1 × 10^7^ cycles, all above as search parameters. The chemical interactions formed in the ligand/receptor complex, were analyzed with Pymol v0.99^35^ molecular viewer and Discovery Studio 2020^36^.

### Physicochemical and ADME-Tox properties analysis and metabolism prediction

Lipinski’s rule of five is a statement of thumb which encompasses several parameters aimed to evaluate, for compounds designed de novo, drug-likeness, or the possibility to be orally administered. Thereby, physicochemical properties of the designed ligands were assessment employing Molinspiration web server (https://www.molinspiration.com/), emphasizing in key attributes of their chemical structures which could affect its water–lipid solubilization and biological activity such as: molecular weight, Log P, h-bond donors and acceptors and total polar surface area (TPSA). Aside, toxic-biological risks that influence on the safety of the designed ligands, were analyzed using Osiris Property Explorer (https://www.organic-chemistry.org/prog/peo/), pointing in such features as mutagenic, tumorigenic, and irritant and reproductive effects. Results yielded of these analyses (physicochemical and ADME-Tox) were included as summation scores for the virtual screening and are included as part of Table S1 on Supplementary Information. Furthermore, metabolism mediated by the CYP450 metabolic enzyme superfamily, was conducted with MetaPrint2D included in Bioeclipse 2.6.2 (https://www.bioclipse.net/metaprint2d), for that, were only included as possible metabolism sites, those contained in the moiety added by the amide bond formation (nucleophilic amines or as appropriate, with possibility to reach the Bcl-2 binding sites). Results yielded of this analysis (metabolism prediction) were included as subtraction scores for the virtual screening and are included as part of Table S1 on Supplementary Information.

### Molecular dynamics simulations with MMGBSA approach.

#### Anchoring of the GPER-ligand into the membrane

Best ligands resulting from VS protocol and subsequently obtained by chemical synthesis, were employed to carried out molecular dynamics simulations, thus, compound **6** and **37** orientation with respect to the bilayer membrane was carried out with OPM (Orientations of Proteins in Membranes) server^37^. The oriented complexes were immersed into a preequilibrated 320 POPC phospholipids bilayer using the membrane-builder tool of CHARM^38,39^ with the following xyz dimensions 110.437 × 110.437 × 122.485 Å. The GPER-ligand-membrane systems were solvated using the TIP3 water model and neutralized with an ionic strength of 0.15 M NaCl.

#### MD simulations

The GPER-ligand-membrane and Bcl2-ligand systems were submitted to 100 ns (ns) MD simulations using the Amber 16 package^40^. Ligand forcefields were constructed considering the Generalized Amber Force Field (GAFF) and the AM1-BCC method. Topology for the GPER-ligand-membrane systems were constructed with a Leap module using ff14SB^41^, Lipid14^42^ and GAFF^43^. Topology for the Bcl2-ligand systems were constructed with a Leap module using ff14SB^41^, and GAFF^43^. MD simulations were performed under periodic boundary conditions using an NPT ensemble at 310 K and 1 bar pressure. Long-range electrostatic interactions were determined with the PME^44^, considering a 10 Å cutoff for van der Waals interactions. Bond lengths were restricted at the equilibrium using the SHAKE algorithm^45^. The temperature was kept using Langevin dynamics. For GPER-ligand-membrane systems the pressure was preserved using a semi-isotropic constant surface tension to maintain the area per lipid. The area per lipid, root means squared deviation (RMSD), radius of gyration (Rg) and clustering examination were carried out using the cpptraj tool in Amber16. Maps of contacts in 2D from the ligand-receptor analysis were made using Maestro Schrödinger version10.5^46^. The Molecular Mechanics Generalized Born Surface Area (MMGBSA)^47^ approach was considered to determine the binding free energy (ΔGbind). The last 40 ns of converged simulation were considered, gathering a total of 4000 snapshots, utilizing implicit solvation models^48^, and a 0.15 M NaCl concentration. The ΔG bind values were determined as previously described^49^.

### Chemical synthesis

All the solvents and reagents were chemical grade obtained from commercial suppliers and employed without further purification. Progress of the reactions were monitored by TLC (thin-layer chromatography) on aluminum-backed sheets with silica gel 60 GF254 (HX805651) and a fluorescent indicator (visualized with UV light of 254 nm). Flash chromatography was performed using silica gel 60 (230–400 mesh) for further purification of the products obtained. Melting points (mp) were determined on an Electrothermal IA 91,000 apparatus (Electrothermal, Bibby Scientific, Staffordshire, ST15 OSA, UK), and are reported as uncorrected. IR spectroscopy was obtained on a Perkin-Elmer 16 FPC FT infrared spectrophotometer and only significant absorption bands are reported as wavenumbers (cm-1) for the representative chemical groups. ^1^H and ^13^C NMR spectra were obtained on a Varian Mercury 300 MHz spectrometer or a Bruker Avance III 750 MHz spectrometer using deuterated chloroform (CDCl3) as the solvent and TMS as the internal standard. Chemical shifts (δ) are reported in ppm downfield from the internal standard and coupling constants are reported in Hertz (Hz) (supplementary material). Electrospray ionization high resolution mass spectrometry positive mode (ESI-HRMS) was performed with an Agilent 6545 QTOF LC/MS instrument (Agilent Technologies, Santa Clara CA, United States).

### Synthesis of (3aS,4R,9bR)-4-(6-bromobenzo[d][1,3]dioxol-5-yl)-3a,4,5,9b-tetrahydro-3H-cyclopenta[c]quinoline-8-carboxylic acid (G1-PABA)

Synthesis of the GPER pharmacophore (tetrahydroquinoline scaffold), named as G1-PABA, was conducted according to the method described by Dennis M.K. et al.^50^, with simple chemical modifications standardized in our work group and described elsewhere^26–28^.

### Synthesis of (3a*S*,4*R*,9b*R*)-4-(6-bromobenzo[*d*][1,3]dioxol-5-yl)-*N*-tosyl-3a,4,5,9b-tetrahydro-3*H*-cyclopenta[*c*]quinoline-8-carboxamide (6) and (3a*S*,4*R*,9b*R*)-4-(6-bromobenzo[*d*][1,3]dioxol-5-yl)-*N*-((4-chlorophenyl)sulfonyl)-3a,4,5,9b-tetrahydro-3*H*-cyclopenta[*c*]quinoline-8-carboxamide (37)

**Figure 1:**
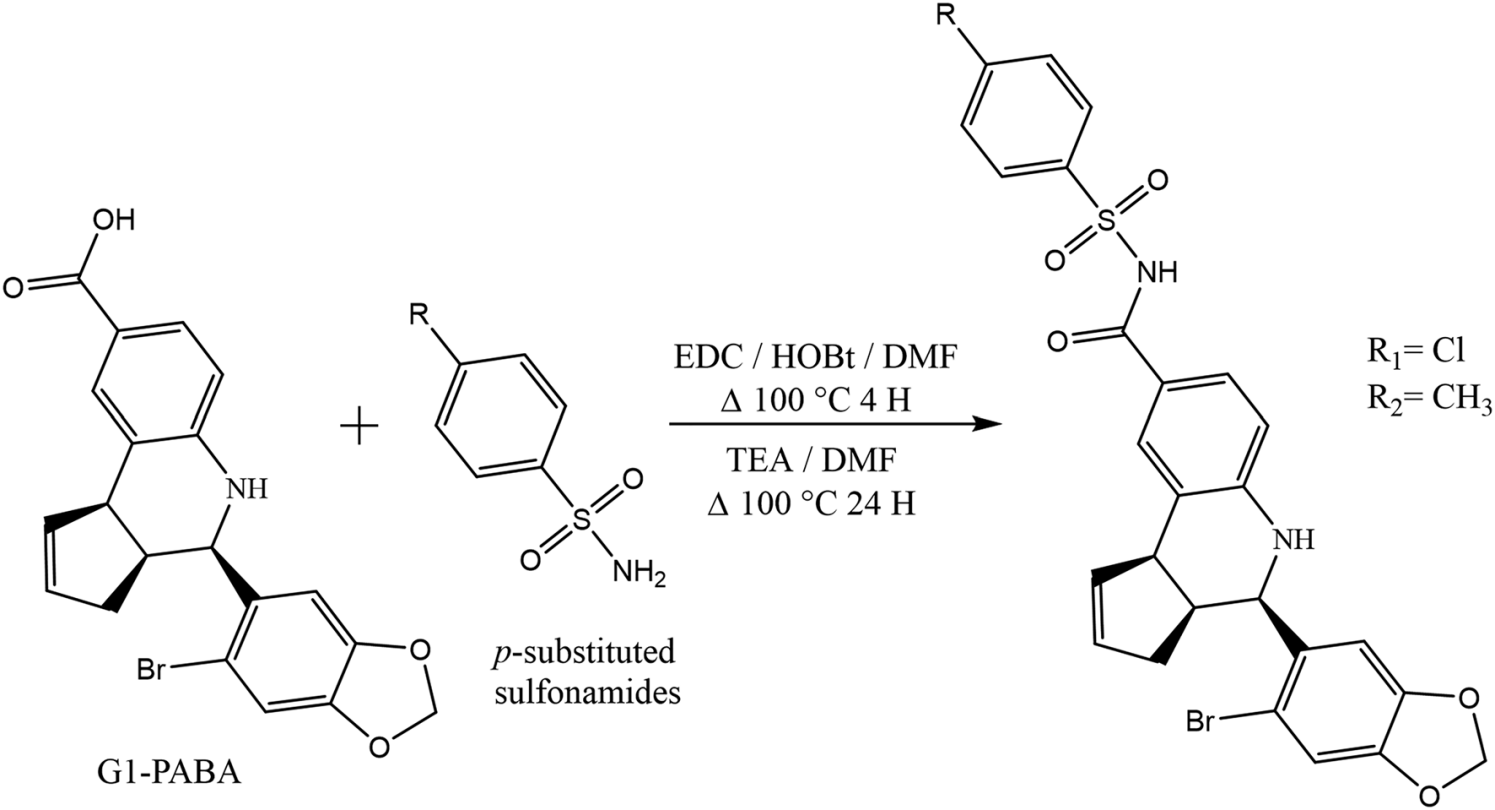
Schematic procedure for the obtention of the tetrahydroquinoline derivatives **6** (R = CH_3_) and **37** (R = Cl) from G1-PABA. Reactants and chemical synthesis conditions are listed up (first step)—down (second step) from the arrow.

A solution of EDC (0.122 g, 0.78 mmol)) and G1-PABA (0.260 g, 0.63 mmol, 1.2 eq) in DMF was added HOBT (0.085 g, 0.63 mmol, 1.2 eq), the reaction was mounted at reflux at 100 °C keeping constant stirring for 4 h. Elapsed this time TEA (0.182 mL, 1.30 mmol, 2.5 eq) was added dropwise and according for the synthesis of **6**, was added 4-methylbenzenesulfonamide (0.100 g, 0.52 mmol, 1 eq) and for the synthesis of **37**, was added 4-chlorobenzenesulfonamide (0.100 g, 0.52 mmol, 1 eq), each reaction mixture was kept at reflux at the previous temperature (100 °C) and constant stirring for a period of 48 h; each reaction were stopped with water and the desired products were extracted with EtOAc (3 × 30 mL) and a final wash with brine, organic layers were dried over Na_2_SO_4_ and concentrated under reduced pressure wit rotary evaporator. Both products were purified by flash chromatography using a mixture of Hexane:EtOAc (7:3) as eluent, affording a 45% yield of compound **6** as a yellow syrup, Rf = 5.2 (Hexane:EtOAc (7:3), mp = (171.3 °C). HPLC purity = 97.90%. Ʋmax: 3313, 2922, 1655, 1602, 1471, 1223, 1033, 659 cm^−1^. ^1^H NMR (750 MHz, CDCl_3_) δ 7.54 ((d, J = 1.3 Hz, H-9), δ 7.38 (dd, J = 8.3, 2.0, H-7), δ 7.12 (d, J = 12.2 Hz, H-6), δ 7.04 (7.04 (d, J = 4.6 Hz, H-3″,5″), δ 6.61 (d, J = 8.4 Hz, H-2″, 6″), δ 6.03—5.99 (d, J = 10.7 Hz, H-1, H-2), δ 5.95–5.92 (d, J = 9.7 Hz, H2′), δ 5.67 (s, H-7′), δ 5.30 (s, H-4′), δ 4.94 (d, J = 3.1 Hz, H-4), δ 4.12 (d, J = 7.3 Hz, H-9b), δ 1.82 (dd, J = 17.3, 8.5 Hz, H-3), 2.54 (dd, J = 15.0, 10.9 Hz, H3), δ 1.25 (CH_3_). ^13^C NMR (189 MHz, CDCl_3_) δ 171.93 (CO), δ 148.34 (C-7′a), δ 147.47 (C-3′a) , δ 146.00 (C-5′), δ 134.13 (C-8), δ 133.86 (C1′′, C-9a), δ 130.41 (C-2), δ 129.47 (C-9), δ 129.05 (C-7), δ 126.53 (C-5′′, C-3′′), δ 126.04 (C-2′′, C-6′′), δ 125.55 (C-6′, C-4′′), δ 115.30 (C-6), δ 113.05 (C-1), δ 112.91 (C-7′), δ 107.97 (C-4′), δ 101.82 (C-2′), δ 56.52 (C-4), δ 45.80 (C-9b), δ 42.10 (C-3a), δ 31.42 (C-3), δ 21.04 (CH_3_). HRMS (ESI) calculated for ([C_27_H_22_BrN_2_O_5_S]): 566.0511. Found: 566.0491. Mass error (ppm): − 3.47. Compound **37** obtained a 40% yield, as a white powder, Rf = 5.4 (Hexane:EtOAc (7:3), mp = (160.7 °C). HPLC purity = 95.30%. Ʋmax: 3350, 2914, 1685, 1608, 1479, 1233, 114, 1037, 831, 704 cm^-1^. ^1^H NMR (750 MHz, CDCl_3_) δ 7.19 (s, H-9), δ 7.13 (s, H-6), δ 7.08 ((dd, J = 8.3, 1.9 Hz, H-7), δ 7.08 (dd, J = 8.3, 1.9 Hz, H-7), δ 7.07 (d, J = 1.9 Hz, H-3″, H-5″), δ 6.59 (d, J = 8.2 Hz, H-2″, 5″), δ 5.99–5.98 (d, J = 9.8 Hz, H2’), δ 5.99 (d, J = 9.8 Hz, H-1, H-2), δ 5.85 (s, H-7′), δ 5.65 (s, H-4′), δ 5.65 (d, J = 4.6 Hz), δ 4.90 (d, J = 3.3 Hz, H-4), δ 4.10 (d, J = 8.6 Hz, H-9b), δ 3.18 (dd, J = 9.0 Hz, 3.3 Hz, H-3a), δ 1.80 (dd, J = 15.6, 8.9 Hz, H-3) δ 2.54 (dd, J = 14.1, 11.8 Hz, H-3). ^13^C NMR (189 MHz, CDCl_3_), δ 171.92 (CO), δ 147.58 (C-7′a), δ 147.38 (C-3′a), δ 146.94 (C-5′), δ 134.13 (C-8), δ 133.86 (C-9a, C1′′), δ 130.42 (C-2), δ 129.05 (C-7, C-9), δ 126.57 (C-5′′, C-3′′), δ 126.05 (C-2′′, C-6′′), δ 125.57 (C-6′, C-4′′), δ 115.29 (C-6), δ 113.06 (C-1), δ 112.92 (C-7′), δ 107.97 (C-4′), δ 101.82 (C-2′), δ 56.53 (C-4), δ 45.80 (C-9b), δ 42.10 (C-3a), δ 31.43 (C-3). The chemical synthesis procedure is depicted in Fig. 1 and NMR ^1^H and ^13^C spectra and HRMS (ESI) data from both compounds are shown in Supplementary Information as Supplementary Figure S1–S6.

### Cell culture

Antiproliferative activity evaluation of the synthesized compounds was assayed in two breast cancer cell lines. Thus, MCF-7 and MDA-MB-231 (American Type Tissue Culture Collection (ATTC), Rockville, MD, USA) were grown in Dulbecco′s Modified Eagle′s Medium (DMEM) media (Life Technologies, Gaithersburg, MD, USA), with 5% FBS (BioWest, Miami, FL, USA), 2 mM glutamine, 100 U/mL penicillin, and 100 mg/mL streptomycin. Cell cultures were incubated at 37 °C in a humidified atmosphere supplemented with 5% CO_2_ and 95% air. Prior to the treatment of the cells, MDA-MB231 and MCF-7 were grown in phenol red-free DMEM media (Life Technologies, Gaithersburg, MD, USA) containing 5% FBS. The FBS was charcoal-stripped (to eliminate the estrogenic effects) only for the MCF-7 cells.

### Cell viability assays

In order to corroborate the possible antiproliferative effect of the synthesized compounds, a MTT [3-(4,5-Dimethyl-2-thiazolyl)-2,5-diphenyl-2H-tetrazolium bromide] assay was carried out. Hence, 1 × 10^4^ cell/well) were cultured in 96-well plates with phenol red-free DMEM media as aforementioned. Synthesized compounds **6** and **37**, and control drugs (Gossypol, a well-known Bcl-2 inhibitor and G-15 an antagonist of GPER), were added at increasing concentrations from 5 to 60 μM, subsequently, the cells were cultured for 72 h. The assay was performed by adding 100 μL of MTT (Sigma, St Louis, MO, USA) (0.25 mg/mL in clear media) to each well, and incubating for 1 h at 37 °C, 5% CO_2_. After the time elapsed, the media was removed, and 200 μL of DMSO (reagent grade) were added to each well to dissolve the formazan crystals. Absorbance was measured at 540 nM in a Mark™ Microplate Absorbance Reader (Bio-Rad Laboratories, Hercules, CA, USA). Each data point was performed in sextuplicate in three different experiments, and the results were reported as the mean absorption ± SD.

## Results and discussion

### Virtual screening converge on two potential GPER and Bcl-2 dual-inhibitors

Results yielded from the series of combined protocols applied to obtain potential inhibitors of GPER and Bcl-2 showed crucial chemical features of the best ranked molecules which are found in other compounds with antiproliferative, and inhibitory growth activity demonstrated mainly in cancer cell lines. Indeed, from the analyzed results, there are a group of molecules with the premise of contain a phenyl sulfonamide moiety as pharmacophore towards GPER and Bcl-2 binding sites and additionally, contains in the aromatic ring of this moiety different *o-*, *m-* and *p-* substituents of electron withdrawing nature as the compounds **6**, **7**, **8**, **9** and **37** (Table S1, column 2), structures in concordance with potent Bcl-2 inhibitors as ABT-737 and ABT-263 which contain a *N*-(phenylsulfonyl)benzamide as pharmacophoric structure^51–53^, highly similar to the structures here designed. Concerning to the mentioned group of phenyl sulfonamide ligands, is worth of mention that, most of them, meet the requirements settled as part of the VS protocol, with only violations in the evaluations encompassed in physicochemical properties, specifically with a Log P and molecular weight slightly increased than is consider optimum according to the Lipinski´s rule of five and, in regards to metabolism prediction which shown a medium probability for ligands **6**, **7**, **8**, and **37**, of being modified in specific atoms of the phenyl sulfonamide moiety, impacting in the final value yielded of the VS protocol. Lastly, as a result of the VS protocol and, according to the availability from the chemical suppliers, just ligands **6** and **37** were obtained by chemical synthesis and evaluated on cell-based assays.

### Analysis of the molecular interactions established in the ligand/receptor complexes

**Figure 2:**
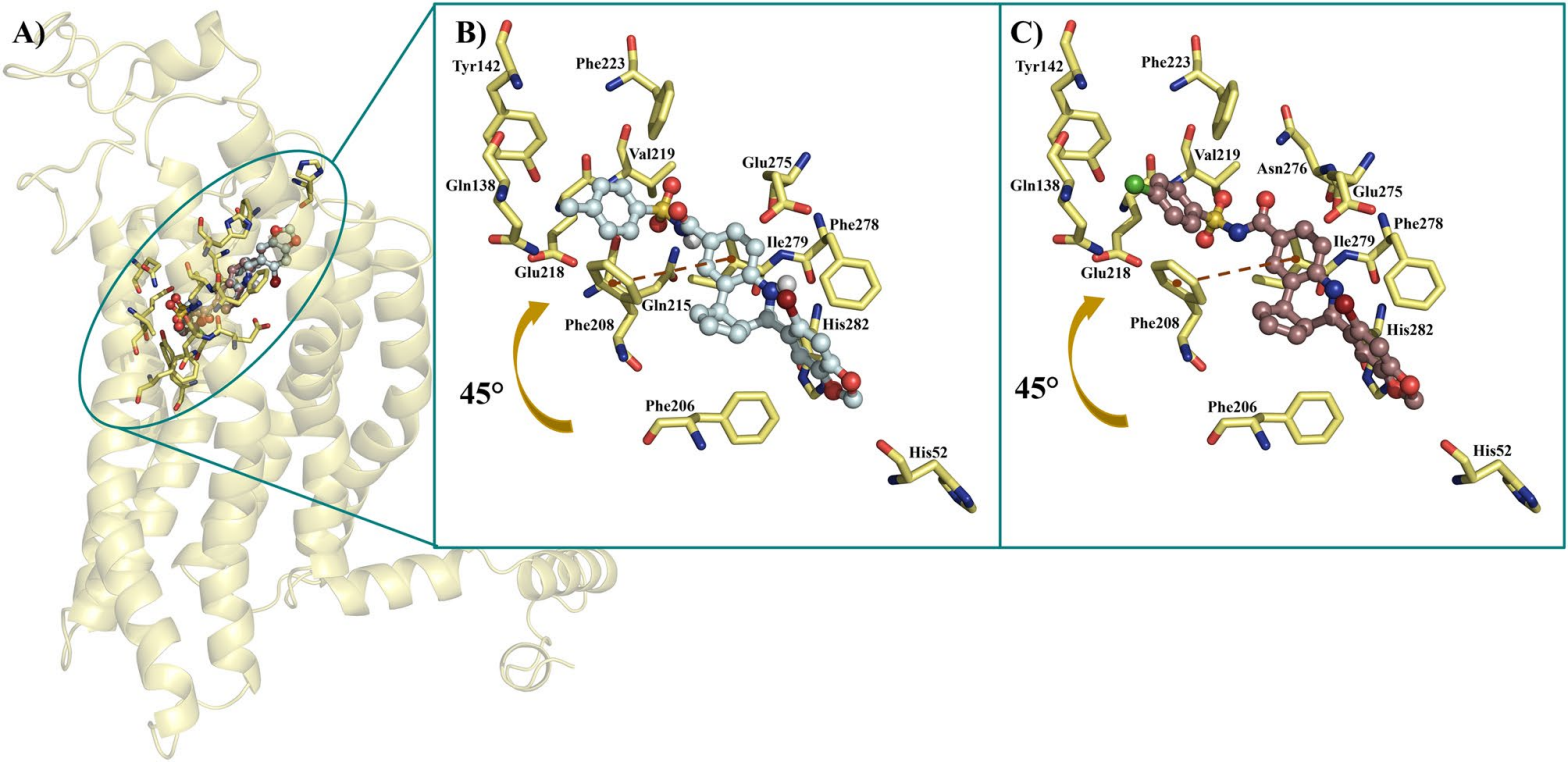
(**A**) Binding modes overlapping of **6** and **37** on GPER binding site. (**B**) Close-up of the **6** and (**C) 37** binding modes. *Pi–pi* interactions are depicted as dark orange dashes and  points a right-turn of 45° of the ligand for a better understanding.

**Figure 3:**
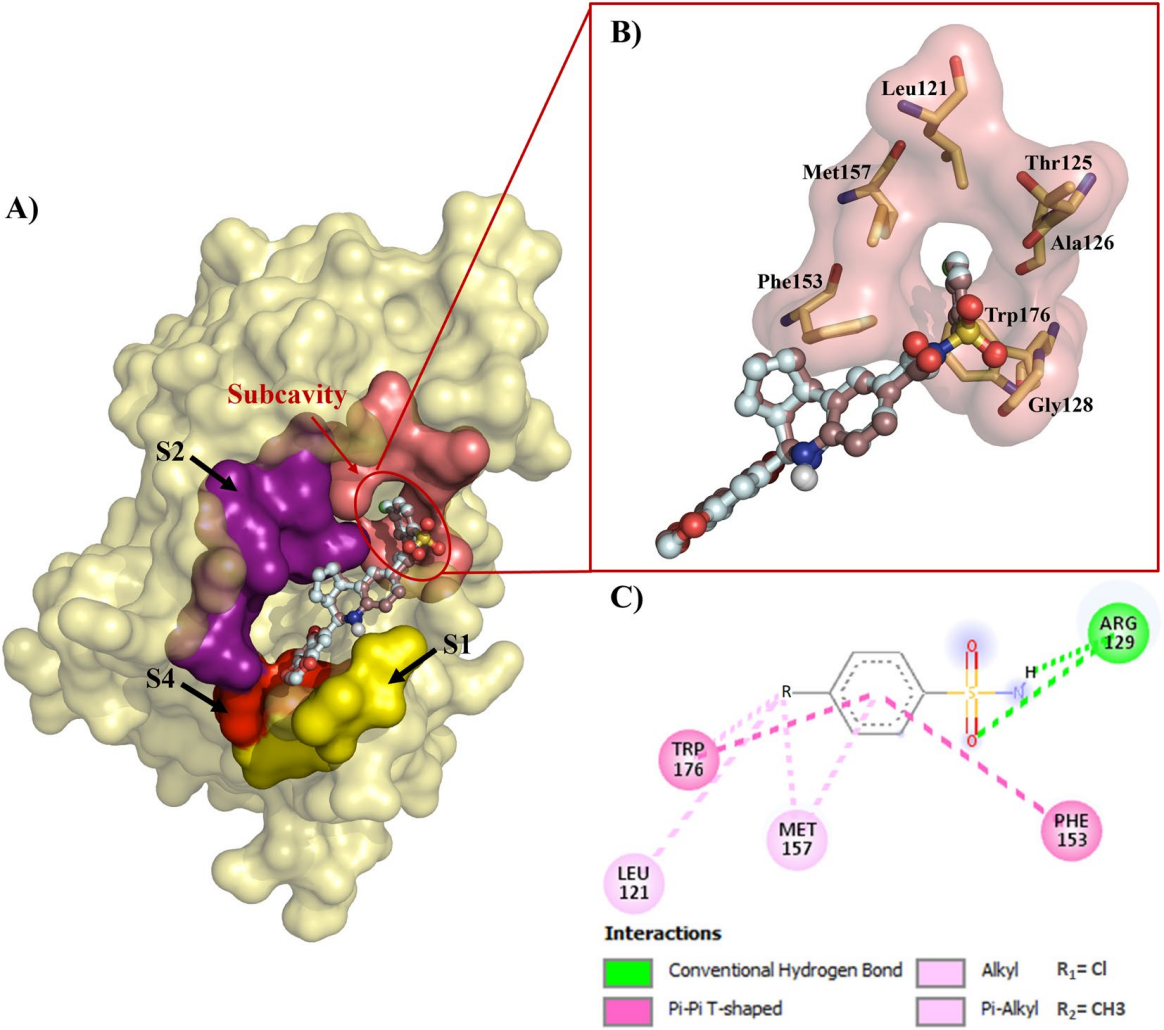
Binding modes overlapping of **6** and **37** on Bcl-2 binding sites. **B)** Isosurface representation of the residues forming the sulfonamide-acceptor subcavity. **C)** 2D map of contacts of the interactions between benzene sulfonamide moiety and the interacting residues.

**Figure 4:**
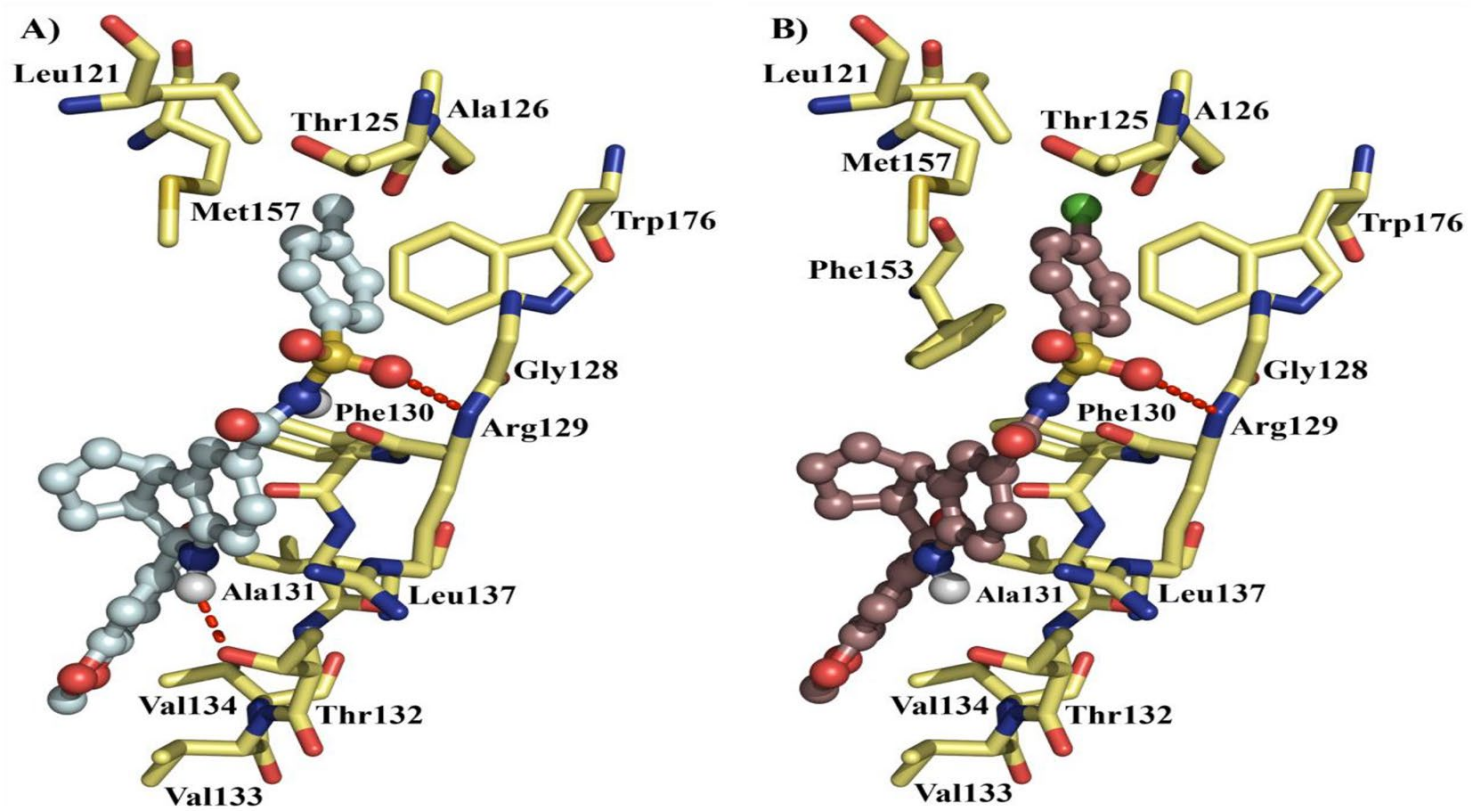
Binding modes of **6** and **37** on Bcl-2. (**A)** Interacting residues for **6** and, (**B)** interacting residues for **37** on the ligand/receptor complex. H-bonds are depicted as red dashes.

As an essential step to reach a total understanding of protein modulation by explicitly designed ligands, the depiction of the chemical interactions established in the ligand/receptor complexes turns into a mandatory requirement for that aim. For such, the molecular docking analysis of the only two synthesized and assayed compounds yield key insights of **6** and **37** regarding reaching the binding sites described for GPER and Bcl-2. Interestingly, both ligands largely share the same binding mode on GPER binding site (Fig. 2A), reaching entirely the phenylalanine cluster formed by Phe206, Phe208, Phe223, and Phe278, described as key orchestrator of the GPER activity^28^. Additionally, the subtle differences among both binding modes lie in the presence of Gln215 establishing a h-bond with one of the oxygen atoms from the sulfonamide moiety of **6** at a close contact of 3.11 Å (Fig. 2B). In contrast, **37** lacks this interaction but establishes contact with Asn276 (Fig. 2C); both binding modes are in an orientation slightly biased to adopt a conformation showed by antagonist compounds of GPER as G15 and G36, which is characterized by modifications on the h-bond occupancy that impacts directly on the helix 2, 3 and 7 rearrangements by establishing interactions with pivotal residues as Phe206, Cys207, Phe208, Phe278 and importantly with Asn310^54^. Furthermore, it is important to note that the phenylalanine cluster stabilizes the ligands mainly through pi-alkyl interactions, with the only exception of Phe278 that forms a dual *pi*–*pi*—T-shaped interaction with the benzene moiety of the bromobenzo dioxol carboxaldehyde from both ligands, contrary to commonly described for agonist ligands in which, is the benzene moiety of the tetrahydroquinoline scaffold of the pharmacophore structure that is establishing a *pi*–*pi* interaction with Phe208 and additionally, reaching the Cys207 to conduct the putative conformational rearrangement between helix 3 and 5 that leads to the activation process of the receptor^54^. Interestingly, **37** depicts no-conventional interactions that arise from the addition of the 4-chloro benzene sulfonamide; in detail, benzene ring of the outlined above moiety, interacts with Glu218 by a *pi*-anion bond and Phe223 with their sulfur atom by a *pi*-sulfur bond, both interactions affecting the binding mode of the ligand on the receptor promoting better binding energy as is possible to note in their ∆G value of − 10.93 kcal/mol. Remarkably, the binding mode overlapping analysis from both ligands converge on a binding site well-defined both for the tetrahydroquinoline scaffold interacting with their benzene moiety by a *pi*-sigma interaction with Ile279 and, additionally, with Phe206 and Phe208 by *pi*-alkyl interactions with the piperidine and cyclopentene ring, consecutively, and lastly with the bromobenzo dioxol carboxaldehyde throughout h-bonds with Gln53 and His282 and the corresponding oxygen atoms of the dioxolane ring and, by a *pi*-alkyl interactions between Ala202 and Pro303 and the carbon atom from the acetal group of the same ring. Regarding the analysis of docking simulations on Bcl-2 protein, results yield encouraging insights to place the synthesized ligands as potential inhibitors similar to BH3 mimetics. Indeed, **6** and **37** reach a binding site bounded by residues in close contact to BH1, and predominantly BH3 domains as Phe130, Ala131, Thr132, Val133, Leu137, Phe153, and Met157 (Fig. 3A), which is the expected for pure BH3 mimetic inhibitors, such as the ligands that deactivate the antiapoptotic family members by filling up their BH3-binding pockets^55,56^, and how is possible to note in the binding mode depicted by Gossypol bounded on Bcl-2 protein, characterized by forming interactions with Asp111, Phe112, Met115, Gln118, Leu119, His120, and very important with the same residues previously described for **6** and **37** as Phe130, Ala131, Thr132, Val133, Leu137, Phe153, Met157^30^. Tellingly, both of the sulfonamide moieties from **6** and **37**, adopt an orientation towards a sulfonamide-acceptor subcavity not described previously and formed by Leu121, Thr125, Ala126, Gly128, Phe153, Met157, and Trp176 (Fig. 3B), this last is the residue most important when interacting both methyl from **6** and chlorine from **37** groups, stabilizing them by an alkyl-chlorine and alkyl-alkyl interactions, respectively, and, for both ligands, by a *pi*–*pi* interaction between the aromatic ring of the sulfonamide moiety and the aromatic ring from the indole group of tryptophan (Fig. 3C). It is worth mentioning that Phe130 and Arg129 are in close contact with both ligands stabilizing them by two h-bonds established between the CO and NH groups from the peptide bond, respectively, and SO and NH from the sulfonamide moiety of the ligands at a distance ranging from 1.71 and 3.02 Å, respectively, placing this residue as a part of a seemingly funnel that connects the sulfonamide moiety with S1, S2 and S4 sub-pockets previously described on Bcl-2 towards ligands that reaches additional binding sites on the protein^51,57^. Intriguingly, Arg129 is an important residue that forms part of the hydrophobic groove present on the surface of the Bcl-2 protein, which, its analogous in Bcl-_XL_ corresponds to a region on the protein with the ability to bind to proapoptotic members of the Bcl-2 family^58,59^, specifically, this structural feature confers to Bcl-_XL_ a higher affinity for Bad and Bak proteins due to a more electronegative state than the Bcl-2 owing to the role of Arg129 to neutralize the electronegativity of the groove. In that sense, it is worth mentioning that **6** and **37** binding modes (Fig. 4A,B), displace the protonable moiety of Arg129, being able to impact its ability to adopt a protonated state modifying its affinity to dimerize with other Bcl-2 family members as Bad and Bak or homodimerizing^60,61^.

### Molecular dynamics simulations results

#### Stability of simulated systems

**Figure 5:**
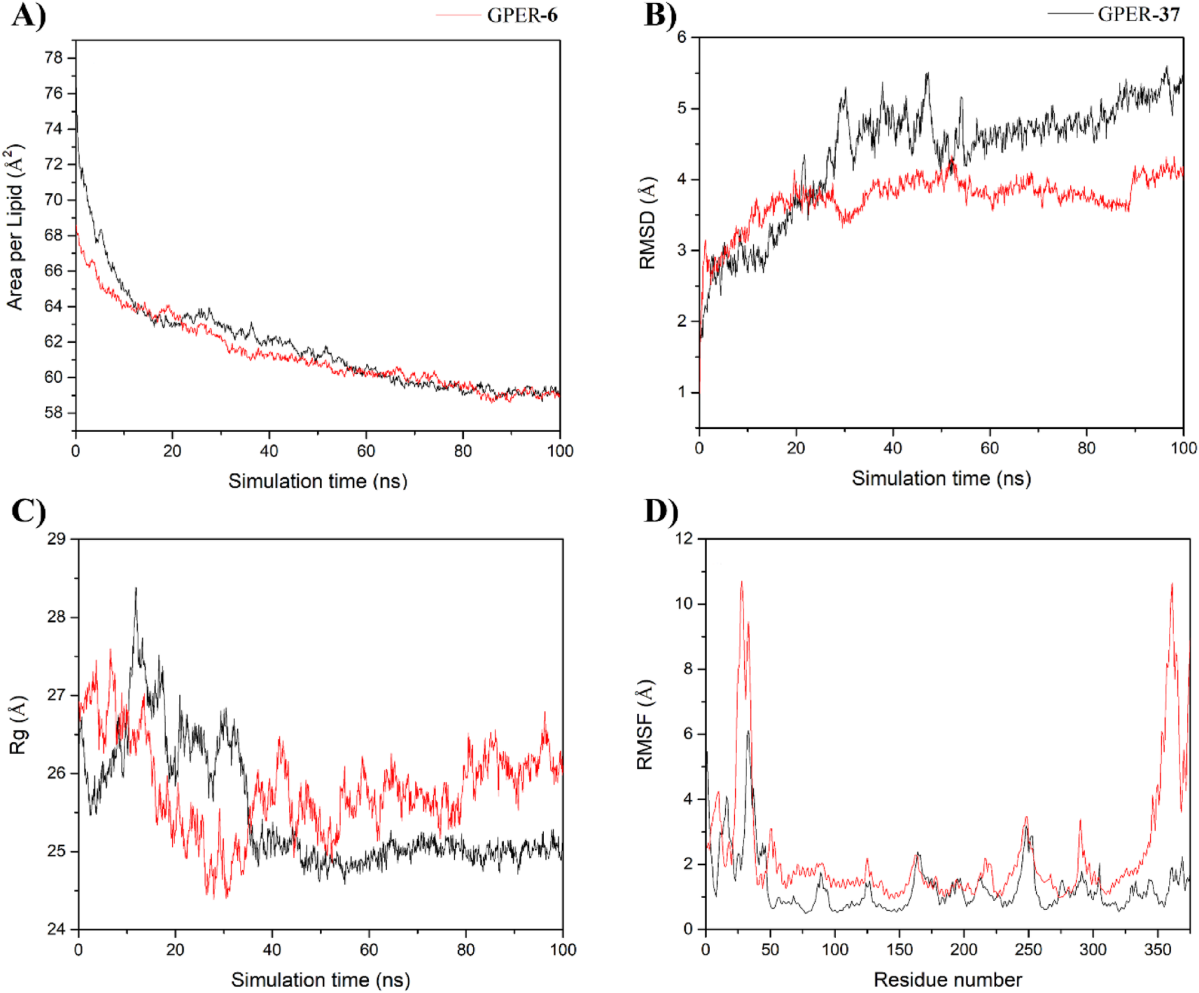
Equilibrium properties of **6** and **37** in complex with GPER through MD simulations. (**A)** Area per lipid, (**B)** Root mean square deviation (RMSD), and (**C)** Radius of gyration (Rg). **6** and **37** behaviors are depicted in black and red lines, respectively.

**Figure 6:**
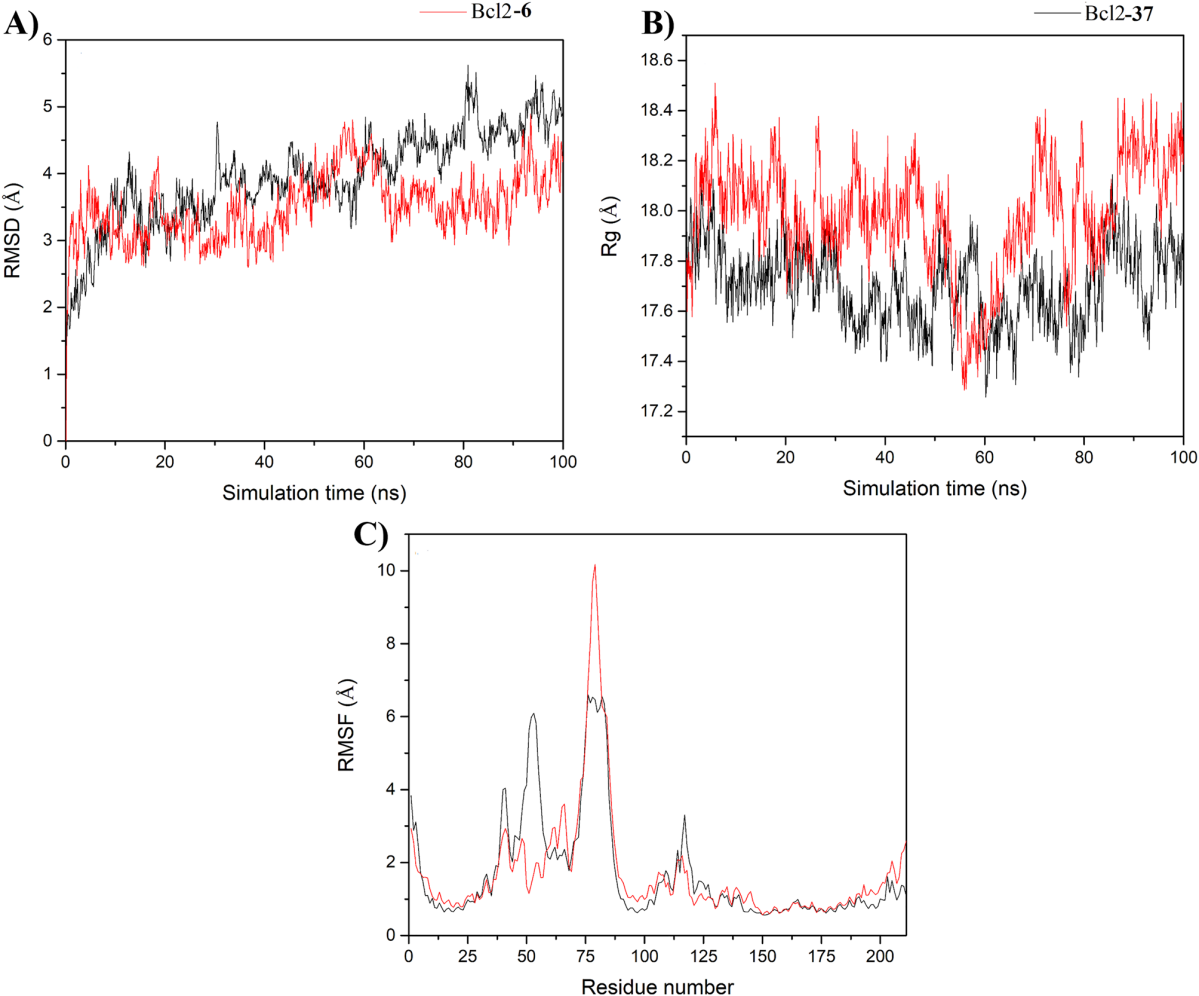
Equilibrium properties of **6** and **37** in complex with Bcl-2 through MD simulations. (**A)** Root mean square deviation (RMSD), and (**B)** Radius of gyration (Rg). **6** and **37** behaviors are depicted in black and red lines, respectively.

The area per lipid, RMSD, and Rg analysis plots display the mobility of the GPER-ligand complexes (Fig. 5), and RMSD and Rg plots show the mobility of Bcl2-ligand systems (Fig. 6). The area per lipid values for GPER-ligand complexes showed convergence between 50 and 60 ns with average values of 59.49 ± 0.40 and 59.5 ± 0.53 for GPER-37 and GPER-6, respectively (Fig. 5A). The GPER-ligand complexes showed RMSD (Fig. 5B) and Rg (Fig. 5C) values that achieved convergence between 20 and 50 ns with average Rg values of 25.0 ± 0.10 and 25.9 ± 0.30 Å for GPER-37 and GPER-6, respectively (Fig. 5C). The Bcl2-ligand complexes showed RMSD (Fig. 6A) and Rg (Fig. 6B) values that equilibrated between 20 and 40 ns with average Rg values of 17.7 ± 0.16 and 18.0 ± 0.20 Å for Bcl2-37 and Bcl2-6, respectively (Fig. 6B). RMSF analysis was determined over the equilibrated simulation time for GPER-ligand complexes (Fig. 5D) showing two high fluctuation peaks corresponding to N-ter and C-ter fragments of both complexes, meanwhile, for Bcl2-ligand complexes (Fig. 6C) there are two regions with increased fluctuations corresponding to regions intrinsically disordered^29,30^. Based on these results, only the last 40 ns and 50 ns were considered for further analysis of GPER-ligand and Bcl2-ligand complexes, respectively.

#### Structural and energetical analysis of receptor-ligand complexes

**Figure 7:**
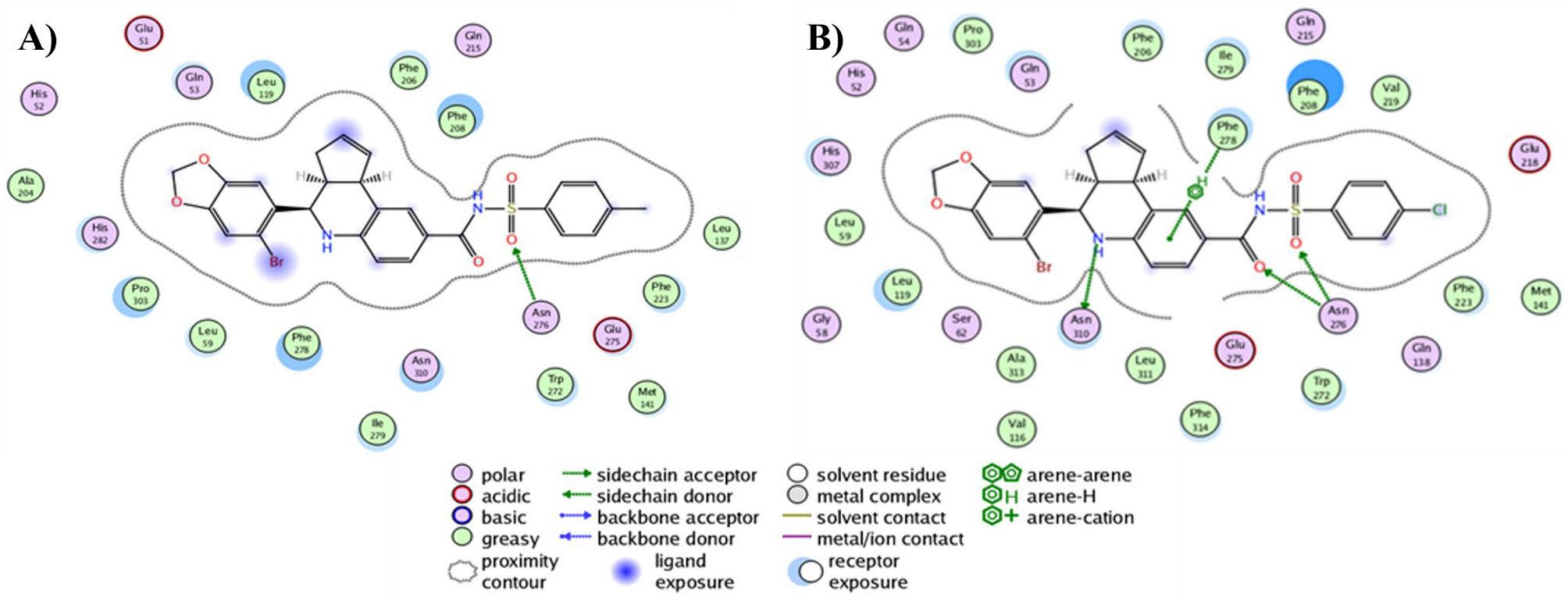
2D Map of contacts of **6** and **37** on GPER. Plot of (**A) 6** and (**B) 37**.

**Figure 8:**
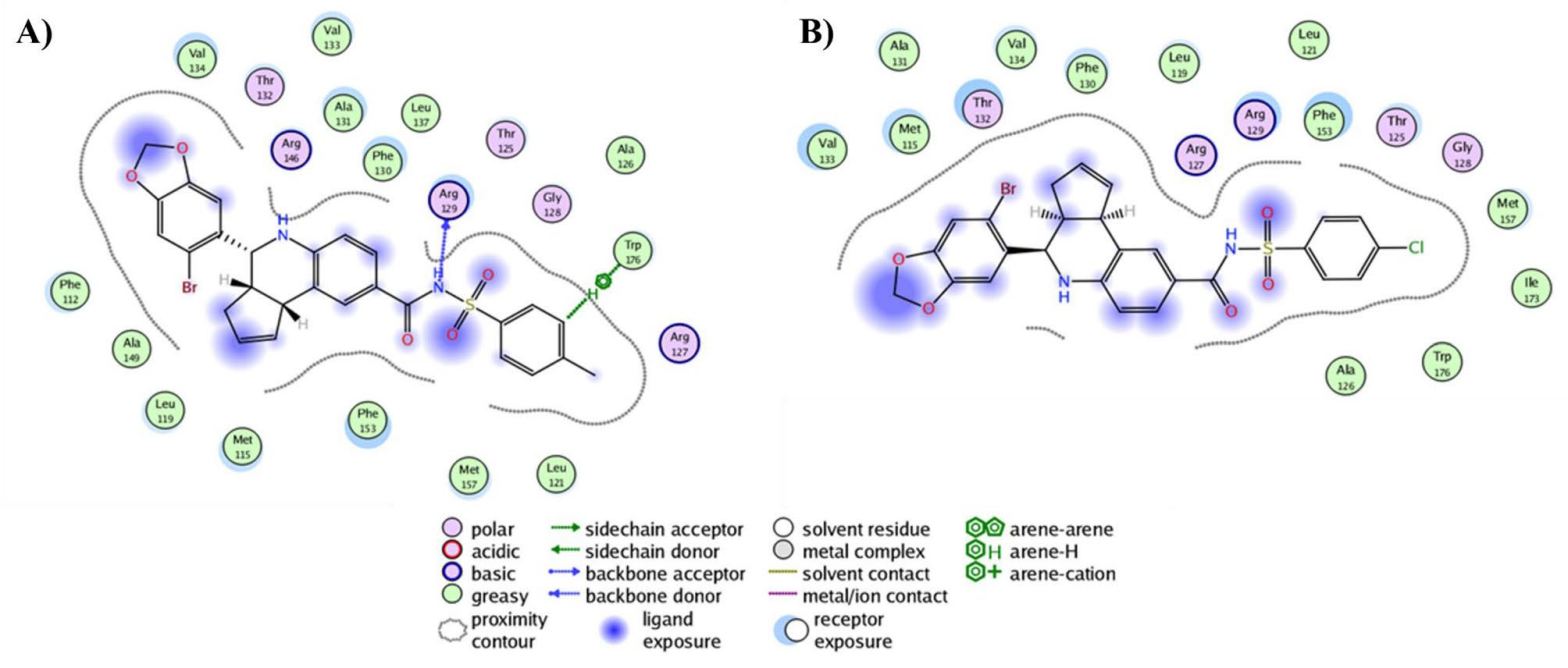
2D Map of contacts of **6** and **37** on Bcl-2. Plot of (**A) 6** and (**B) 37**.

Representative GPER-ligand (Fig. 7) and Bcl2-ligand (Fig. 8) conformations were obtained through cluster analysis, considering a cut-off of 2.5 Å. Compound **37** on GPER is coordinated by a series of residues in which the main type of interactions is hydrophobic established between residues Leu59, Val116, Leu119, Met141, Phe206, Phe208, Val219, Phe223, Trp272, Phe278, Ile279, Pro303, Leu311, Phe314, and Ala313 with the compound. (Fig. 7A). Noteworthy, Phe206, Phe208, Phe223, and Phe278 residues that form the phenylalanine cluster into GPER are interacting with the ligand mainly through pi-alkyl interactions, detailing, is Phe208 and Phe278, the residues that confer an improved stabilization to the ligand/receptor complex, owing to non-polar interactions between the aromatic ring of F208 and the chlorine atom and the pentadiene ring of **37**, whilst, Phe278 interacts through a pi–pi interaction with the aromatic ring and with a pi-alkyl with the piperidine ring, both from the tetrahydroquinoline moiety of **37**, respectively. Additionally, this compound formed polar contacts with His52, Gln53, Gln54, Gly58, S62, Gln138, Gln215, Glu218, Glu275, and His307. Remarkably, from the hydrogen bonds established between side chains of Asn276 and Asn310 with **37**, it is interesting that sulfonamide moiety of the ligand is stabilized by two h-bonds provided by Asn276 at a distance lesser than 3 Å. In regard to compound **6**, it is bound by most of the same interacting residues and through non-polar interactions than **37**, as it is Leu59, Leu119, Leu137, Met141, Ala204, Phe206, Phe208, Phe223, Trp272, Phe278, Ile279, and Pro303. Strikingly, is the phenylalanine cluster that stabilizes compound **6** through a binding mode very similar to that depicted for compound **37**, with the main stabilizing interactions provided by Phe208 and Phe278. Besides, the polar interactions correspond to those given by Glu51, His52, Gln53, Gln215, Glu275, Asn276, His282, and Asn310. Similarly analyzed for compound **37**, Asn276 is the residue stabilizing the sulfonamide moiety of **6** by forming two hydrogen bonds (Fig. 7B). Besides, analysis of Bcl-2/ligand complexes yielded structural insights into the ability of both compounds to reach and maintain key interactions with residues closed to subpockets previously described as binding sites on Bcl-2 protein. Thus, compound **37** by 12 hydrophobic residues: Met115, Leu119, Val133, Leu121, Ala126, Phe130, Ala131, Val134, Phe153, Met157, Ile173, and Trp176. And 5 polar residues: Thr125, Gly128, Arg127, Arg129, and Thr132 (Fig. 8A), it is worth emphasizing that several residues previously listed are encompassed into BH1-BH3 domains, which are the proteinic target of pure BH3 mimetics inhibitors. Moreover, a striking find of the MD results is the maintaining of the sulfonamide-acceptor subcavity formed by Leu121, Thr125, Ala126, Gly128, Arg129, Phe153, Met157 and Trp176, with the inclusion now of Ile173 as an additional residue stabilizing along the time to compound **37** by forming an alkyl interaction with the chlorine atom from the sulfonamide moiety of this ligand, further interactions towards stabilize this moiety are established by *pi*-alkyl and *pi-pi* interactions provided by Met157, and Phe153 and Trp176, respectively, at an average distance lesser to 5 Å. A last detail regarding to polar interactions, is the pivotal role of Arg129 to forms a funnel that connects some residues from the S1, S2 and S4 subpockets on Bcl-2 with the sulfonamide-acceptor subcavity owing to the formation of two h-bonds at 2.74 and 2.96 Å with the CO and SO from the sulfonamide bond of **37**, respectively. Finally, compound **6** on the Bcl2-ligand complex shown a binding mode very similar to the depicted for **37**, with slight differences as far as the sulfonamide-acceptor subcavity, in detail, Met157, Phe153 and Trp176 are found establishing the same interactions with the chloro-phenyl moiety than **37**, with the only exception that Ile173 is lacking in the structural analysis. Additionally, total description of the interactions established in the receptor-ligand complex shown that **6** was coupled by 14 hydrophobic residues: Phe112, Met115, Leu119, Leu121, Ala126, Phe130, Ala131, Val133, Val134, Leu137, Ala149, Phe153, Met157, and Trp176 (Fig. 8B). Polar interactions were taken by Thr125, Arg127, Gly128, Arg129, Thr132 and Arg146. Arg129 also formed one hydrogen bond with the sulfonamide bond of **6**, very similar to the described for the **37** and the apparently funnel formed by this residue. Lastly, comparative analysis revealed that **37** reached a higher number of interactions with GPER than **6**. In contrast, is this last compound that showed a more optimized map of receptor-ligand interactions for Bcl-2 than **37**.

#### Binding Free Energy decomposition of receptor-ligand complexes

The binding affinity of the ligands with GPER or Bcl-2 was determined over the equilibrate simulation time using the MMGBSA method. Table 1 shows that the two compounds exhibited favorable binding free energies on GPER or Bcl2. Comparative analysis between the different contributions to the binding free energy (ΔG_bind_) points that the Van der Waals energy (ΔE_vdw_) was the major force stabilizing the ligand on both receptors and, specifically for Bcl-2, the chemical nature of **6** and **37** containing in both structures *pi* electrons able to interact throughout *pi*-alkyl interactions with most of the residues from the groove contained on Bcl-2 protein, might affecting directly on the displacement that suffer Arg129 (on its protonable moiety) and in the affinity for dimerize with proapoptotic proteins or homodimerization. This analysis suggests a more favorable binding affinity of **6** to Bcl-2 than GPER. Whereas a similar affinity was observed for **37** on both receptors.

**Table 1:** Binding free energy components of GPER or Bcl-2 in complexes with **6** and **37** (kcal/mol).

System	*ΔE*_*vdw*_	*ΔE*_*ele*_	*ΔG*_*ele,sol*_	*ΔG*_*npol,sol*_	*ΔG*_*bind*_
GPER-**6**	− 59.83 ± 4.0	− 28.88 ± 5.0	54.98 ± 3.0	− 7.85 ± 0.4	− 41.52 ± 4.0
GPER-**37**	− 57.69 ± 3.3	− 15.24 ± 5.3	36.3 ± 4.0	− 6.8 ± 0.40	− 43.5 ± 4.0
Bcl2-**6**	− 62.87 ± 4.0	− 18.69 ± 4.0	41.01 ± 4.0	− 6.92 ± 0.4	− 47.50 ± 4.0
Bcl2-**37**	− 54.62 ± 3.0	− 15.42 ± 3.8	33.45 ± 3.0	− 5.96 ± 0.2	− 42.56 ± 3.0

### Cell proliferation analysis

**Figure 9:**
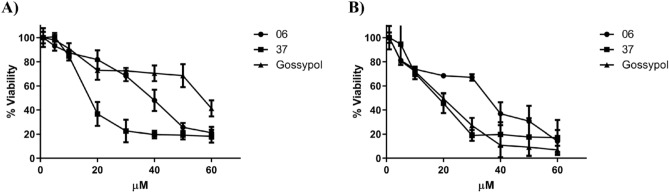
Effect of **6** and **37** on cell viability of breast cancer cells. (**A)** MCF-7 and (**B)** MDA-MB-231. Each data point was performed in sextuplicate in three different experiments, and the results were reported as the mean absorption ± SD.

MCF-7 cells and MDA-MB-231 cell were subjected to treatments with **6** and **37** to corroborate these compounds’ ability to inhibit cell proliferation. Outlined above, MCF-7 (ER and PR ( +) and MDA-MB-231 (ER, PR, and HER2 (−) cell lines were considered as models of the most common and aggressive subtype of breast cancer according to their molecular expression pattern, in addition to a deregulated Bcl-2 activity promoting an anti-apoptotic effect^62,63^. As is depicted in Fig. 9, in MCF-7 both compounds showed a better performance, measured as a decrease in the percentage of cell viability, than Gossypol, in a concentration-dependent manner without involving the cell number seeded; this finding is an outstanding insight into the probable mechanism of action conducted by **6** and **37** which points mainly to a probable Bcl-2 inhibition, reinforced for a GPER modulation, since, from the energetical analyzes yielded by in silico simulations over the two pharmacological targets, the affinity of both compounds is slightly more representative for GPER in comparison with Bcl-2 (< onefold), however, from the individual results analysis, **37** decreased cell proliferation in a better way than **6** probably due to the slightly finer binding energy of this ligand towards Bcl-2 as is possible to contrast it both docking calculations and molecular dynamics simulations. Astoundingly, MDA-MB-231 cancer cell were more sensitive to the assayed compounds than MCF-7 cells, owing largely to a over expression of Bcl-2 protein on MDA-MB-231 cell line^64,65^, added to a slight GPER expression that in a basal state, triggers proliferation and cancer cell survival^66,67^, although, from the individual results obtained for MCF-7 cancer cells, **37** depicted an IC_50_ value significantly lower than Gossypol (18.66 vs. 65.67 μM, respectively), probably due to multiple effects of this ligand at inhibit both estrogen receptors (ERs α > β and GPER) and the anti-apoptotic protein Bcl-2, triggering a synergistic effect which leads to a best antiproliferative performance than showed by Gossypol, besides, we were unable to determine the IC_50_ value for G-15, due to solubilization problems starting at a 20 μM concentration (Data not showed). The highlighted above place both compounds as potential therapeutic options against most aggressive cancer type TNCB (Triple-Negative Breast Cancer) represented by the two cancer cell lines assayed. IC_50_ values of **6** and **37** assayed on MDA-MB-231 and MCF-7 cancer cell lines are collected on Table 2.

**Table 2:** IC_50_ values of **6** and **37** on the cancer cell lines assayed.

Compound	IC_50_ µM	
	*MDA-MB-231*	*MCF-7*
Gossypol	16.67	65.67
6	29.54	37.03
37	17.31	18.66

## Conclusions

Nowadays, BC disease including its most aggressive form (TNBC), represents a challenge for new drug design. Hence, bioinformatic tools are a fundamental piece aimed at finding targeted compounds focused on blockade novel pharmacological targets involved in proliferation and survival, as are GPER and Bcl-2. In this work, we show the antiproliferative activity assayed on MDA-MB-231 (a TNBC cell model) of **37**, a contained chlorine-benzene sulfonamide compound, with a very encouraging IC_50_ value of 17.31 μM, closed to the one of Gossypol (16.67 μM) a well-know Bcl-2 inhibitor. Moreover, computational simulations yield intriguing insights about the capability of compounds **6** and **37** to reach the binding sites of GPER and Bcl-2, characterized by a mandatory interaction with the pivotal phenylalanine cluster of GPER, and with the BH3-BH1 domains, respectively, with proper binding energies computed throughout MD simulations including values ranging from − 41.52 to − 43.50 kcal/mol, and − 42.56 to − 47.50 kcal/mol, for each simulated protein, respectively. Accordingly, these findings place **6** and **37** as potential pharmacological therapeutic options for use against the most aggressive breast cancer type (TNBC).

### Supplementary Information

Supplementary Information.

## Data Availability

The datasets generated and analyzed during the current study are available from the corresponding authors upon reasonable request.

## References

[1] Doi K (2012). Discovery of marinopyrrole A (maritoclax) as a selective Mcl-1 antagonist that overcomes ABT-737 resistance by binding to and targeting Mcl-1 for proteasomal degradation. J. Biol. Chem..

[2] Hanahan D, Weinberg RA (2011). Hallmarks of cancer: The next generation. Cell.

[3] Adams JM, Cory S (2007). The Bcl-2 apoptotic switch in cancer development and therapy. Oncogene.

[4] Roberts AW, Huang D (2017). Targeting BCL2 with BH3 mimetics: Basic science and clinical application of venetoclax in chronic lymphocytic leukemia and related B cell malignancies. Clin. Pharmacol. Ther..

[5] Buolamwini JK (1999). Novel anticancer drug discovery. Curr. Opin. Chem. Biol..

[6] Ochi K, Mori T, Toyama Y, Nakamura Y, Arakawa H (2002). Identification of semaphorin3B as a direct target of p53. Neoplasia.

[7] Kirkin V, Joos S, Zornig M (2004). The role of Bcl-2 family members in tumorigenesis. Biochim. Biophys. Acta.

[8] Park CM (2006). Design, synthesis, and computational studies of inhibitors of Bcl-XL. J. Am. Chem. Soc..

[9] Tse C (2008). ABT-263: A potent and orally bioavailable Bcl-2 family inhibitor. Cancer Res..

[10] Soderquist R, Bates DJ, Danilov AV, Eastman A (2013). Gossypol overcomes stroma-mediated resistance to the BCL2 inhibitor ABT-737 in chronic lymphocytic leukemia cells ex vivo. Leukemia.

[11] Yu T (2020). GPER mediates decreased chemosensitivity via regulation of ABCG2 expression and localization in tamoxifen-resistant breast cancer cells. Mol. Cell Endocrinol..

[12] Yu T (2014). GPER mediates enhanced cell viability and motility via non-genomic signaling induced by 17beta-estradiol in triple-negative breast cancer cells. J. Steroid. Biochem. Mol. Biol..

[13] Chan YT (2020). GPER-induced signaling is essential for the survival of breast cancer stem cells. Int. J. Cancer.

[14] Magruder HT (2014). The G protein-coupled estrogen receptor-1, GPER-1, promotes fibrillogenesis via a Shc-dependent pathway resulting in anchorage-independent growth. Horm. Cancer.

[15] Rouhimoghadam M, Lu AS, Salem AK, Filardo EJ (2020). Therapeutic perspectives on the modulation of G-protein coupled estrogen receptor, GPER, function. Front. Endocrinol. (Lausanne).

[16] Ramsay RR, Popovic-Nikolic MR, Nikolic K, Uliassi E, Bolognesi ML (2018). A perspective on multi-target drug discovery and design for complex diseases. Clin. Transl. Med..

[17] Lu JJ, Pan W, Hu YJ, Wang YT (2012). Multi-target drugs: The trend of drug research and development. PLoS ONE.

[18] Quinn JA (2009). Coordinate regulation of estrogen-mediated fibronectin matrix assembly and epidermal growth factor receptor transactivation by the G protein-coupled receptor, GPR30. Mol. Endocrinol..

[19] Marjon NA, Hu C, Hathaway HJ, Prossnitz ER (2014). G protein-coupled estrogen receptor regulates mammary tumorigenesis and metastasis. Mol. Cancer Res..

[20] Bianchini G, Balko JM, Mayer IA, Sanders ME, Gianni L (2016). Triple-negative breast cancer: Challenges and opportunities of a heterogeneous disease. Nat. Rev. Clin. Oncol..

[21] Chapdelaine AG, Sun G (2023). Challenges and opportunities in developing targeted therapies for triple negative breast cancer. Biomolecules.

[22] Yang R (2022). Therapeutic progress and challenges for triple negative breast cancer: Targeted therapy and immunotherapy. Mol. Biomed..

[23] Rosales-Hernandez MC, Correa-Basurto J (2015). The importance of employing computational resources for the automation of drug discovery. Expert Opin. Drug Discov..

[24] Hernandez-Rodriguez M, Rosales-Hernandez MC, Mendieta-Wejebe JE, Martinez-Archundia M, Basurto JC (2016). Current tools and methods in molecular dynamics (MD) simulations for drug design. Curr. Med. Chem..

[25] Bello M, Martinez-Archundia M, Correa-Basurto J (2013). Automated docking for novel drug discovery. Expert Opin. Drug Discov..

[26] Martinez-Munoz A (2018). Selection of a GPER1 ligand via ligand-based virtual screening coupled to molecular dynamics simulations and its anti-proliferative effects on breast cancer cells. Anticancer Agents Med. Chem..

[27] Zacarias-Lara OJ (2019). Synthesis and In vitro evaluation of tetrahydroquinoline derivatives as antiproliferative compounds of breast cancer via targeting the GPER. Anticancer Agents Med. Chem..

[28] Mendez-Luna D (2021). Modifications on the tetrahydroquinoline scaffold targeting a phenylalanine cluster on GPER as antiproliferative compounds against renal. Liver and pancreatic cancer cells. Pharmaceuticals (Basel).

[29] Ilizaliturri-Flores I (2016). Mapping the intrinsically disordered properties of the flexible loop domain of Bcl-2: A molecular dynamics simulation study. J. Mol. Model.

[30] Zacarias-Lara OJ, Correa-Basurto J, Bello M (2016). Exploring the conformational and binding properties of unphosphorylated/phosphorylated monomeric and trimeric Bcl-2 through docking and molecular dynamics simulations. Biopolymers.

[31] ACD/ChemSketch v. 1.2 (Advanced Chemistry Development, Inc., Toronto, ON, Canada, 2020).

[32] Gauss View v. 5 (Semichem Inc., Shawnee Mission, 2009).

[33] Gaussian 09 v. Revision A.02 (Gaussian, Inc., Wallingford CT, 2016).

[34] Morris GM (2009). AutoDock4 and AutoDockTools4: Automated docking with selective receptor flexibility. J. Comput. Chem..

[35] The PyMOL Molecular Graphics System (DeLano Scientific, San Carlos, CA, USA, 2002).

[36] Discovery Studio 2022 (BIOVIA, San Diego: Dassault Systèmes, 2022).

[37] Lomize AL, Pogozheva ID, Lomize MA, Mosberg HI (2006). Positioning of proteins in membranes: A computational approach. Protein Sci..

[38] Trott O, Olson AJ (2010). AutoDock Vina: Improving the speed and accuracy of docking with a new scoring function, efficient optimization, and multithreading. J. Comput. Chem..

[39] Jojart B, Martinek TA (2007). Performance of the general amber force field in modeling aqueous POPC membrane bilayers. J. Comput. Chem..

[40] Case DA (2005). The Amber biomolecular simulation programs. J. Comput. Chem..

[41] Maier JA (2015). ff14SB: Improving the accuracy of protein side chain and backbone parameters from ff99SB. J. Chem. Theory Comput..

[42] Dickson CJ (2014). Lipid14: The amber lipid force field. J. Chem. Theory Comput..

[43] Wang J, Wolf RM, Caldwell JW, Kollman PA, Case DA (2004). Development and testing of a general amber force field. J. Comput. Chem..

[44] Darden T, York D, Pedersen L (1993). Particle mesh Ewald: An N⋅log(N) method for Ewald sums in large systems. J. Chem. Phys..

[45] van Gunsteren WF, Berendsen HJC (1977). Algorithms for macromolecular dynamics and constraint dynamics. Mol. Phys..

[46] Maestro v. 10.5 (Schrödinger, LLC, New York, NY).

[47] Wang E (2019). End-point binding free energy calculation with MM/PBSA and MM/GBSA: Strategies and applications in drug design. Chem. Rev..

[48] Feig M (2004). Performance comparison of generalized born and Poisson methods in the calculation of electrostatic solvation energies for protein structures. J. Comput. Chem..

[49] Bello M (2018). Binding mechanism of kinase inhibitors to EGFR and T790M, L858R and L858R/T790M mutants through structural and energetic analysis. Int. J. Biol. Macromol..

[50] Dennis MK (2009). In vivo effects of a GPR30 antagonist. Nat. Chem. Biol..

[51] Oltersdorf T (2005). An inhibitor of Bcl-2 family proteins induces regression of solid tumours. Nature.

[52] Wendt MD (2006). Discovery and structure-activity relationship of antagonists of B-cell lymphoma 2 family proteins with chemopotentiation activity in vitro and in vivo. J. Med. Chem..

[53] Petros AM (2006). Discovery of a potent inhibitor of the antiapoptotic protein Bcl-xL from NMR and parallel synthesis. J. Med. Chem..

[54] Mendez-Luna D, Bello M, Correa-Basurto J (2016). Understanding the molecular basis of agonist/antagonist mechanism of GPER1/GPR30 through structural and energetic analyses. J. Steroid Biochem. Mol. Biol..

[55] Soderquist RS, Eastman A (2016). BCL2 inhibitors as anticancer drugs: A plethora of misleading BH3 mimetics. Mol. Cancer Ther..

[56] Tutumlu G (2020). Integrating ligand and target-driven based virtual screening approaches with in vitro human cell line models and time-resolved fluorescence resonance energy transfer assay to identify novel hit compounds against BCL-2. Front. Chem..

[57] Nagy MI (2021). Design, synthesis, anticancer activity, and solid lipid nanoparticle formulation of indole- and benzimidazole-based compounds as pro-apoptotic agents targeting Bcl-2 protein. Pharmaceuticals (Basel).

[58] Murray JB (2019). Establishing drug discovery and identification of hit series for the anti-apoptotic proteins, Bcl-2 and Mcl-1. ACS Omega.

[59] Sattler M (1997). Structure of Bcl-xL-Bak peptide complex: Recognition between regulators of apoptosis. Science.

[60] Zhang Z (2004). Bcl-2 homodimerization involves two distinct binding surfaces, a topographic arrangement that provides an effective mechanism for Bcl-2 to capture activated Bax. J. Biol. Chem..

[61] Lee EF (2019). Structural insights into BCL2 pro-survival protein interactions with the key autophagy regulator BECN1 following phosphorylation by STK4/MST1. Autophagy.

[62] Jo EH (2007). Induction of apoptosis in MCF-7 and MDA-MB-231 breast cancer cells by Oligonol is mediated by Bcl-2 family regulation and MEK/ERK signaling. Eur. J. Cancer Prev..

[63] Giordano F (2023). Valproic acid inhibits cell growth in both MCF-7 and MDA-MB231 cells by triggering different responses in a cell type-specific manner. J. Transl. Med..

[64] Ferenc P, Solar P, Kleban J, Mikes J, Fedorocko P (2010). Down-regulation of Bcl-2 and Akt induced by combination of photoactivated hypericin and genistein in human breast cancer cells. J. Photochem. Photobiol. B.

[65] Maarouf RE, Azab KS, El Fatih NM, Helal H, Rashed L (2023). Withania somnifera Alter BCL2/Bax signaling and trigger apoptosis of MCF-7 and MDA-MB231 breast cancer cells exposed to gamma-radiation. Hum. Exp. Toxicol..

[66] Pereyra-Vergara F, Olivares-Corichi IM, Perez-Ruiz AG, Luna-Arias JP, Garcia-Sanchez JR (2020). Apoptosis induced by (-)-epicatechin in human breast cancer cells is mediated by reactive oxygen species. Molecules.

[67] Hsu LH, Chu NM, Lin YF, Kao SH (2019). G-protein coupled estrogen receptor in breast cancer. Int. J. Mol. Sci..

