# Supplementary material for: In silico design and cell-based evaluation of two dual anti breast cancer compounds targeting Bcl-2 and GPER
Source: Sci Rep. 2023 Oct 20;13:17933. doi: 10.1038/s41598-023-43860-x (PMC10589355; doi:10.1038/s41598-023-43860-x)
Supplement: Supplementary file 1 — Supplementary Information. [file 41598_2023_43860_MOESM1_ESM.docx]

**Table S1. Results from the Virtual Screening protocol. |BE| (Binding Energy in absolute value), from the interacting residues columns the value next to the sign of + indicates the total of the summative score, LogP (Logarithm of partition), MW (Molecular Weight), nON (Number of H-bond acceptors), nOHNH (Number of H-bond donnors). For a detailed explanation of each header column see VS methodology.**

| **ID** | Nucleophilic amine moiety | BE on Bcl-2 (kcal/mol) | BE on GPER (kcal/mol) | Interacting residues from Bcl-2 by molecular docking | Interacting residues from GPER by molecular docking | Molinspiration results | | Osiris Property Explorer results | Metaprint results | FINAL |
| --- | --- | --- | --- | --- | --- | --- | --- | --- | --- | --- |
| **1** |  | -10.1 | -10.1 | D64, A67, T69, S70, P71, Q73, Q92, H94, L95, R207, L209, F210, D211  0+ | I107, A110, D111, I114, N118, L129, F132, F136, V139, N140, W182  0+ | LogP  MW  nON  nOHNH | 5.57 1034.09 13 4 | MUT: 0  TER: 10  IRRIT: 0  EF REP: 0 | -25 | -4.9 |
| **2** |  | -10.04 | -9.71 | K22, Q25, R26, S105, R106, R107, TYR108, R109, F112, A113, S117, Q118, E152, Q159, E160, N163, R164  0+ | I107, D111, N115, L129, F132, F136, N140, G179, W182, M183, V186  0+ | LogP  MW  nON  nOHNH | 8.31  896.93  10  2 | MUT:10  TER:10  IRRIT: 10  EF REP: 10 | -5 | 64.7 |
| **3** |  | -11.03 | -10.15 | E13, K17, P75, P78, G83, P84, P90, P91, Q92, H94, L95, R207, L209, F210  0+ | Y234, L241, R259, L262, A263, V265, L266, F264, V270, P274, V309  0+ | LogP  MW  nON  nOHNH | 8.72  952.91  11  3 | MUT: 10  TER: 10  IRRIT: 10  EF REP: 10 | -15 | 45.15 |
| **4** |  | -9.36 | -9.10 | F112, E114, M115, Q118, L119, H120, T125, R127, G128, R129, F153, M157  20+ | I107, I114, N118, F132, F136, V139, N140, W182, V186  0+ | LogP  MW  nON  nOHNH | 7.03  805.48  11  2 | MUT: 10  TER: 10  IRRIT: 0  EF REP: 0 | -30 | 9.1 |
| **5** |  | -10.25 | -9.51 | M115, H120, L121, L124, T125, A126, R127, R129, F130, A131, Q133, Q134, L137, F153, M157, W176  40+ | I107, I114, N118, F132, F136, V139, N140, W182, V186  0+ | LogP  MW  nON  nOHNH | 6.03  777.67  10  2 | MUT: 10  TER: 10  IRRIT: 10  EF REP: 0 | -55 | 4.5 |
| **6** |  | -10.5 | -11.02 | L121, T125, A126, G128, R129, F130, A131, Y132, V133, V134, L127, M157, W176  0+ | H52, Q138, Y142, F206, F208, Q215, E128, Y219, F223, E275, F278, I279, H282  91+ | LogP  MW  nON  nOHNH | 5.33  567.46  7  2 | MUT: 10  TER: 10  IRRIT: 10  EF REP: 10 | -20 | 142.76 |
| **7** |  | -10.45 | -11.58 | F112, M115, G128, R129, F130, T132, Q133, Q134, F153  30+ | Q53, Q54, G58, L119, F206, C207, F208, I279, R286, P303, G306, H307, N310  75+ | LogP  MW  nON  nOHNH | 5.45  632.88  10  2 | MUT: 10  TER: 10  IRRIT: 10  EF REP: 10 | -10 | 136.58 |
| **8** |  | -10.42 | -10.86 | D10, E13, I14, K17, G83, L86, P90, P91 H94  0+ | Q53, Q54, G58, L59, F208, F223 W272, E275, N276, F278, I279, P303, G306, H307, N310, F314  58+ | LogP  MW  nON  nOHNH | 4.89  553.43  7  2 | MUT: 10  TER: 10  IRRIT: 10  EF REP: 10 | -10 | 128.86 |
| **9** |  | -10.6 | -11.91 | D111, F112, M115, L119, T125, R129, F130, L137, R146, F153, A149  20+ | Q53, Q54, G58, L119, F206, C207, F208, I279, R286, P303, G306, H307, N310  75+ | LogP  MW  nON  nOHNH | 4.84  598.43  1  0  2 | MUT: 10  TER: 10  IRRIT: 10  EF REP: 10 | 0 | 156.91 |
| **10** |  | -9.48 | -11.45 | R98, Q99, D102, M206, P208, L209, F210  0+ | Q138, Y142, F208, E218, E275, N276, F278, I279, P303, G306, N310  25+ | LogP  MW  nON  nOHNH | 4.52  598.43  10  2 | MUT:10  TER: 10  IRRIT: 10  EF REP: 0 | -10 | 86.45 |
| **11** |  | -9.03 | -10.45 | D111, F112, L119, T125, R129, F130, L137, R146, F153  20+ | Q138, Y142, F208, E218, E275, N276, F278, I279, P303, G306, N310  25+ | LogP  MW  nON  nOHNH | 5.20  505.37  6  3 | MUT: 10  TER: 10  IRRIT: 10  EF REP: 10 | -5 | 90.45 |
| **12** |  | -9.15 | -10.18 | Y9, D10, E13, I14, K17, G83, P84, L86, P90, P91, H94  0+ | Q53, L119, L137, Q138, M141, Y142, F208, E218, V219, F223, E275, F314  16+ | LogP  MW  nON  nOHNH | 5.63  575.46  7  2 | MUT: 10  TER: 10  IRRIT: 10  EF REP: 10 | -30 | 56.18 |
| **13** |  | -9.16 | -10.82 | L119, H120, L121, T125, R129, F130, A131, T132, Q133, F134, L137, F153, M157  30+ | L108, S112, L137, M141, Y142, F208, Q215, E218, F223, W272, E275, N276, I279, A313, N316, S317  0+ | LogP  MW  nON  nOHNH | 4.95  527.41  7  2 | MUT: 10  TER: 10  IRRIT: 10  EF REP: 10 | -30 | 50.82 |
| **14** |  | -9.7 | -10.32 | F112, L119, H120, L121, T125, R129, F130, A 131, T132, Q133, Q134, L137, F153, M157  30+ | Q53, L119, L137, Q138, Y142, F208, E218, V219, F223, W272, E275, N310, F314  25+ | LogP  MW  nON  nOHNH | 5.63  575.46  7  2 | MUT: 10  TER: 10  IRRIT: 10  EF REP: 10 | -30 | 65.32 |
| **15** |  | -8.9 | -11.11 | D10, E13, I14, K17, T74, P75, P78, G83, P84, P90, P91, H94  0+ | Q53, L119, L137, Q138, Y142, F208, E218, V219, F223, W272, E275, N310, F314  25+ | LogP  MW  nON  nOHNH | 5.23  547.40  7  2 | MUT: 10  TER: 10  IRRIT: 10  EF REP: 0 | -25 | 61.11 |
| **16** |  | -9.04 | -9.92 | F112, M115, L119, H120, R129, F130, A131, T132, Q134, F153, M157  30+ | F227, I230, G231, Y234, V238, L241, R259, L262, A263, V265, L266, F269, L327  0+ | LogP  MW  nON  nOHNH | 6.53  722.64  9  3 | MUT: 10  TER: 10  IRRIT: 10  EF REP:10 | -55 | 14.92 |
| **17** |  | -10.05 | -8.84 | G8, Y9, D10, N11, E13, G83, L86, P90, N182, H186, Q190  0+ | F227, G231, Y234, L241, R259, L262, A263, V265, L266, F264  0+ | LogP  MW  nON  nOHNH | 7.34  801.53  9  3 | MUT: 10  TER: 10  IRRIT: 10  EF REP: 10 | -50 | 18.84 |
| **18** |  | -9.59 | -10.28 | Y9, D10, E13, I14, K17, G83, L86, P90, P91, H94, F210  0+ | Q138, Y142, F208 E218, E275, N276, F278, I279, G306, N310  16+ | LogP  MW  nON  nOHNH | 5.67  489.37  5 | MUT: 10  TER: 10  IRRIT: 10  EF REP: 10 | -15 | 81.28 |
| **19** |  | -9.75 | -10.7 | L119, H120, L121, T125, R129, F130, A131, T132, Q134, L137, F153, M157  30+ | Q138, Y142, F208, E218, E275, N276, F278, I279, G306, N310  16+ | LogP  MW  nON  nOHNH | 5.54  517.42  5  2 | MUT: 10  TER: 10  IRRIT: 0  EF REP: 10 | -20 | 56.7 |
| **20** |  | -9.66 | -10.11 | D111, M115, L119, L121, T125, R129, T132, T132, Q134, L137, F153, M157  10+ | Q53, Q138, Y142, F208, E218, E275, N276, F278, I279, P303, G306, N310  25+ | LogP  MW  nON  nOHNH | 5.58  549.38  8  4 | MUT: 10  TER: 10  IRRIT: 10  EF REP: 10 | -25 | 70.11 |
| **21** |  | -9.72 | -10.15 | D10, E13, I14, K17, G83, L86, P90, P91, H94, F210  0+ | L108, S112, L137, F206, F208, W272, E275, F278, I279, R,286, A313, F314  8+ | LogP  MW  nON  nOHNH | 5.68  519.39  6  2 | MUT: 10  TER: 10  IRRIT: 0  EF REP: 10 | -20 | 48.15 |
| **22** |  | -9.7 | -10.69 | Y9, D10, E13, I14, K17, G83, L86, P90, P91, H94, F201  0+ | Q54, G58, L59, S62, F206, F208, E275, N276, F278, I279, H282, P303, N310  50+ | LogP  MW  nON  nOHNH | 6.08  503.40  5  2 | MUT: 10  TER: 10  IRRIT: 5  EF REP: 10 | -15 | 100.69 |
| **23** |  | -9.72 | -10.46 | D10, E13, I14, K17, G83, L86, P90, P91, H94, F210  10+ | Q138, Y142, F208, E218, E275, N276, F278, I279, P303, G306, N310  16+ | LogP  MW  nON  nOHNH | 6.55  557.37  5  2 | MUT: 10  TER: 10  IRRIT: 10  EF REP: 10 | -5 | 81.46 |
| **24** |  | -11.13 | -10.89 | L119, H120, L121, T125, R129, F130, A131, T132, Q133, Q134, L137, F153, M157  30+ | L108, S112, F206, F208, W272, E275, F278, I279, R286, A313, F314, S317  16+ | LogP  MW  nON  nOHNH | 5.61  534.37  8  2 | MUT: 10  TER: 10  IRRIT: 10  EF REP: 10 | -5 | 81.89 |
| **25** |  | -9.11 | -10.88 | M1, F104, R107, Y108, N143, W144, G145, R146, Q148, W188, N192, Y202  0+ | Q138, Y142, F208, E218, F223, E275, N276, F278, P303, G306, N310  25+ | LogP  MW  nON  nOHNH | 5.58  531.41  6  2 | MUT: 0  TER: 10  IRRIT: 10  EF REP: 10 | -10 | 75.88 |
| **26** |  | -9.77 | -10.58 | M1, F104, R107, Y108, N143, W144, G145, R146, Q148, W188, Y20  0+ | Q138, Y142, F208, E218, F223, E275, N276, F278, P303, G306 H308, N310  25+ | LogP  MW  nON  nOHNH | 5.63  534.37  8  2 | MUT: 10  TER: 10  IRRIT: 10  ER :10 | 0 | 95.58 |
| **27** |  | -8.69 | -10.36 | D111, F112, L119, L121, T125, R129, F130, L137, R146, A149, F153, M157  20+ | Q138, Y142, F208, E218, E275, N276, F278, I279, F303, G306, H307, N310  33+ | LogP  MW  nON  nOHNH | 5.73  519.39  6  2 | MUT: 10  TER: 10  IRRIT: 10  EF REP: 10 | - 25 | 78.36 |
| **28** |  | -9.02 | -10.74 | M1, F104, R107, Y108, N143, W144, G145, R146, Q148, W188, N192, Y202  0+ | Q53, Q54, G58, L59, S62, L119, F223, E275, N276, F278, P303, N310  58+ | LogP  MW  nON  nOHNH | 6.48  568.26  5  2 | MUT: 10  TER: 0  IRRIT: 10  EF REP:10 | -15 | 103.74 |
| **29** |  | -8.95 | -10.57 | D34, Q35, Q52, P53, T56, P57, H58, P59  0+ | Q53, Q54, G58, L59, S62, L119, F223, E275, N276, F278, P303, N310  58+ | LogP  MW  nON  nOHNH | 6.35  523.81  5  2 | MUT: 10  TER: 10  IRRIT: 10  EF REP: 10 | 0 | 128.57 |
| **30** |  | -8.86 | -10.63 | D10, E13, I14, K17, P75, A76, P78, G83, L86, P90, P91, H94,  0+ | Q138, Y142, F208, E218, E275, N276, F278, I279, P303, G306, H307, N310  33+ | LogP  MW  nON  nOHNH | 6.12  503.40  5  2 | MUT: 10  TER: 10  IRRIT: 10  EF REP: 10 | -20 | 83.63 |
| **31** |  | -9.83 | -10.62 | D10, E13, I14, K17, L86, P90, P91, H94, F210  0+ | Q138, Y142, F208, E218, E275, N276, F278, I279, G306, N310  16+ | LogP  MW  nON  nOHNH | 6.10  503.40  5  2 | MUT: 10  TER: 10  IRRIT: 10  EF REP: 10 | -15 | 71.6 |
| **32** |  | -9.22 | -10.28 | D111, F112, L119, L121, R129, F130, L137, A149, F153, M157  20+ | L108, S112, M141, F206, F208, W272, E275, F278, I279, R286, S317  16+ | LogP  MW  nON  nOHNH | 5.54  521.39  5  2 | MUT: 10  TER: 10  IRRIT: 10  EF REP: 10 | -5 | 81.28 |
| **33** |  | -9.28 | -10.08 | M1, F104, R107, Y108, N143, W144, G145, R146, Q148, W188, N192, Y202,  0+ | Q138, Y142, F208, E218, F223, E275, N276, F278, P303, G306, H307, N310  33+ | LogP  MW  nON  nOHNH | 5.59  533.38  7  3 | MUT: 10  TER: 10  IRRIT: 10  EF REP: 10 | -25 | 78.08 |
| **34** |  | -9.1 | -10.36 | D10, E13, I14, K17, G83, L86, P90, P91, H94, F210  0+ | Q138, Y142, F208, E218, E275, N276, F278, I279, P303, G306, N310  25+ | LogP  MW  nON  nOHNH | 5.84  507.36  5  2 | MUT: 10  TER: 10  IRRIT: 10  EF REP: 10 | -20 | 75.36 |
| **35** |  | -9.8 | -10.98 | D10, E13, I14, K17, P75, A76, P78, G83, L86, P90, P91, H94  0+ | Q53, Q54, G58, L59, S62, L119, F206, F223, E275, F278, I270, P303, N310  50+ | LogP  MW  nON  nOHNH | 6.76  615.26  5  2 | MUT: 10  TER: 10  IRRIT: 10  EF REP: 10 | -10 | 110.98 |
| **36** |  | -9.59 | -9.99 | Y9, D10, E13, I14, K17, G83, L86, P90, P91, H94, F210  0+ | Q138, Y142, F208, E218, Q275, N276, F278, I279, G306, N310  16+ | LogP  MW  nON  nOHNH | 5.91  525.35  5  2 | MUT: 10  TER: 10  IRRIT: 10  EF REP: 10 | -15 | 70.99 |
| **37** |  | -10.87 | -10.93 | L121, T125, A126, G128, R129, F130, A131, T132, V133, V134, L137, F153, M157, W176  50+ | H52, Q138, Y142, F206, F298, E218, V219, F223, E275, N276, F278, I279, H282  66% | LogP  MW  nON  nOHNH | 5.56  587.88  7  2 | MUT: 10  TER: 10  IRRIT: 10  EF REP: 10 | -15 | 121.53 |
| **38** |  | -8.97 | -9.96 | L119, H120, L121, T125, R129, F130, A131, T132, Q133, Q134, L137, F153, M157  30+ | H52, Q53, Q54, G58, F206, F208, F278, I279, H282, R286, P303, H307, N310  66% | LogP  MW  nON  nOHNH | 2.10  485.33  7  3 | MUT: 10  TER: 10  IRRIT: 10  EF REP: 10 | -5 | 150.96 |
| **39** |  | -10.9 | -8.86 | L119, H120, L121, T125, R129, F130, A131, T132, L137, F153, M157  30+ | Y 42, F208, Q215, E218, E219, F223, E275, F 278, I279, G306, H307, N310  16+ | LogP  MW  nON  nOHNH | 3.41  527.41  7  3 | MUT: 10  TER: 10  IRRIT: 10  EF REP: 10 | -10 | 84.4 |
| **40** |  | -9.45 | -8.69 | F112, L119, H120, L121, T125, R129, F130, A131, T132, L137, A149, F153, M157  30+ | Q53, Q54, G58, L119, F206, F208, F278, I279, H282, R286, P303, G306, H307, N310  83+ | LogP  MW  nON  nOHNH | 2.55  545.46  7  3 | MUT: 10  TER: 10  IRRIT: 10  EF REP: 10 | -20 | 141.69 |
| **41** |  | -10.0 | -9.48 | Y 9, D10, E13, I14, K17, L86, P90, P91, H94, W95  0+ | N84, R88, E89, K90, M91, L96, Y97, I99, N100, F147, H173 S177, W182  0+ | LogP  MW  nON  nOHNH | 3.56  561.43  7  3 | MUT: 10  TER: 10  IRRIT: 10  EF REP: 10 | -5 | 74.48 |
| **42** |  | -9.83 | -10.93 | F112, M115, T125, G128, R129, F130, F153  20+ | Y142, F208, Q215, E218, V219, F223, E275, F278, I279, G306, H307, N310  25+ | LogP  MW  nON  nOHNH | 3.71  600.47  8  4 | MUT: 10  TER: 10  IRRIT: 10  EF REP: 10 | -5 | 100.93 |
| **43** |  | -10.01 | -8.5 | D10, E13, I14, K17, L86, P90, P91, H94, L95, F210  0+ | H52, Q53, F208, F223, E275, I279, F278, H282, P303, N310  33+ | LogP  MW  nON  nOHNH | 3.65  541.44  7  3 | MUT: 10  TER: 10  IRRIT: 10  EF REP: 10 | -15 | 96.5 |
| **44** |  | -10.35 | -10.59 | D111, F112, M115, G128, R129, F130, T132, A149, F153, W176  40+ | Q53, Q138, M141, F206, Q215, E218, V219, F223, E275, N276, I279  8+ | LogP  MW  nON  nOHNH | 4.37  640.33  7  3 | MUT: 10  TER: 10  IRRIT: 10  EF REP: 10 | -5 | 83.59 |
| **45** |  | -10.38 | -10.48 | Y9, D10, N11, R12, E13, I14, K17, L86, P90, P91, H94, H186  0+ | F227, A228, G231, Y234, L241, R259, L262, V265, L266, F269, V270, P274  0+ | LogP  MW  nON  nOHNH | 6.68  803.69  13  3 | MUT: 10  TER: 10  IRRIT: 10  EF REP: 10 | -5 | 55.48 |
| **46** |  | -11.04 | -10.37 | F112, M115, L119, H120, T125, G128, R129, F130, A131, T132, Q133, Q134, L137, F153, M157  30+ | L96, Y97, N100, V103, I107, F136, N140, F147, H173, S177, W182  0+ | LogP  MW  nON  nOHNH | 6.35  780.05  12  3 | MUT: 10  TER: 10  IRRIT: 10  EF REP: 10 | -15 | 45.37 |
| **47** |  | -9.95 | -12.71 | D111, F112, M115, L119, H120, L121, G128, R129, F130, L137, A149, F153, M157  30+ | Q53, G58, L59, Q 38, M141, Y142, F208 E218, V219, F223, E275, F278, P303, G306, N310  41+ | LogP  MW  nON  nOHNH | 5.78  700.61  9  3 | MUT: 10  TER: 10  IRRIT: 10  EF REP: 10 | -15 | 97.71 |
| **48** |  | -11.49 | -10.6 | T7, G8, Y9, D10, N11, E13, I14, G83, L86, S87, P90, N182, H186, Q190  0+ | M91, Y97, I99, N100, V103, F136, N140, F147, S177, W182  0+ | LogP  MW  nON  nOHNH | 8.00  886.18  13  3 | MUT: 10  TER: 10  IRRIT: 10  EF REP: 10 | -25 | 35.6 |
| **49** |  | -10.03 | -10.3 | D10, E13, I14, P78, A82, G83, P84, L86, P88, P90, P91, Q92, H94, L95, F210  0+ | W150, F153, D154, I157, H172, L176, T220, L221, V225, P226, A228, I 229, L232  0+ | LogP  MW  nON  nOHNH | 7.28  838.13  13  3 | MUT: 10  TER: 10  IRRIT: 10  EF REP: 10 | -25 | 35.3 |
| **50** |  | -10,87 | -9.8 | A42, A43, L119, F124, T125, R127, G128, R129, F153, M157, N172  20+ | K256, R259, A263, L319, L322, I323, F326, L327, R332  0+ | LogP  MW  nON  nOHNH | 7.96  931.17  16  3 | MUT: 10  TER: 10  IRRIT: 10  EF REP: 10 | -25 | 34.8 |
| **51** |  | -11.72 | -11.86 | F112, M115, Q118, L119, H120, T125, G128, F130, A131, T132, Q133, Q134, F153, M157  30+ | L81, V82, N84, I85, Y97, I99, N199, V103, F147, H173, L176, S177, I181, W182  0+ | LogP  MW  nON  nOHNH | 6.81  819.09  13  4 | MUT: 10  TER: 10  IRRIT: 10  EF REP: 10 | -15 | 46.86 |
| **52** |  | -9.87 | -10.59 | F112, M115, L119, H120, L121, T125, F130, A131, T132, Q133, Q134, F153, M157  20+ | Q53, Q54, G58, L59, S112, E115, L137, F208, W272, E275, F278, P303, G306, N310, F314  50+ | LogP  MW  nON  nOHNH | 5.63  666.59  9  3 | MUT: 10  TER: 10  IRRIT: 10  EF REP: 10 | -15 | 105.59 |
| **53** |  | 10.07 | -10.13 | Y9, D10, N11, E13, G83, L86, S87, P90, N182, H186, Q190  0+ | L81, N84, I99, N100, I107, F136, N140, F147, L176, S177, G179, I181, W182  0+ | LogP  MW  nON  nOHNH | 6.69  753.67  10  4 | MUT: 10  TER: 10  IRRIT: 10  EF REP: 10 | -5 | 65.13 |
| **54** |  | -11.07 | -10.18 | F112, M115, Q118, L119, H120, T125, F130, A131, T132, Q133, Q134, F153, M157  20+ | I 107, A110, D111, I114, M133, F136, L137, V139, N140, W182  0+ | LogP  MW  nON  nOHNH | 6.59  779.51  9  3 | MUT: 10  TER: 10  IRRIT: 10  EF REP: 10 | -15 | 55.18 |
| **55** |  | -10.57 | -10.57 | L119, H120, T125, R127, G128, R129, F130, A131, F138, F153, M157, W176, Y180  70+ | I107, D111 E115, L129, F132, M133, F136, N140, G179, W182, M183, V186  0+ | LogP  MW  nON  nOHNH | 8.46  968.90  12  4 | MUT: 10  TER: 10  IRRIT: 10  EF REP: 10 | -10 | 50.57 |
| **56** |  | -9.81 | -10.28 | Y9, D10, E13, I14, K17, P75, A76, P78, G83, P84, L86, P90, P91, H94, D211  0+ | L81, L96, Y97, I99, N100, V03, N 140, F147, S177, C178, G179, I181, W182  0+ | LogP  MW  nON  nOHNH | 8.99  933.95  11  2 | MUT: 10  TER: 10  IRRIT: 10  EF REP: 0 | -10 | 40.28 |
| **57** |  | -10.43 | -11.08 | K17, H20, Y21, S24, A67, S70, P71, Q73, P75, R98, F210, D211  0+ | F146, T149, W150, F153, H 172, L176, A184, A 88, L221, V225, P226, I229  0+ | LogP  MW  nON  nOHNH | 9.17  973.93  11  2 | MUT: 10  TER: 10  IRRIT: 10  EF REP: 0 | -10 | 41.08 |
| **58** |  | -11.8 | -12.14 | D10, E13, I14, K17, G83, P84, L86, P90, P91, H94, L95, L209, F210  0+ | L12, F146, T149, W150, L176, I181, A184, S187, A188, V191, P192, L217, T220, L221, V225, P226, I229  0+ | LogP  MW  nON  nOHNH | 9.23  972.94  10  2 | MUT: 10  TER: 10  IRRIT: 10  EF REP: 10 | -20 | 52.14 |
| **59** |  | -10,99 | -10.27 | L119, H120, L121, T125, G128, R129, F130, A131, T132, Q133, Q134, L137, F153, M157, W176  40+ | L106, I107, A110, D111, I114, N118, L129, F132, M133, F136, L137, N140  0+ | LogP  MW  nON  nOHNH | 9.00  989.93  12  3 | MUT: 10  TER: 10  IRRIT: 10  EF REP: 0 | -10 | 40.27 |
| **60** |  | -9.81 | -10.35 | K17, H20, Y21, A67, S70, P75, G83, P90, P91, H94, F210, D211  0+ | V103, I104, D111, I114, L129, F132, M133, F136, L137, V139, N140, W182  0+ | LogP  MW  nON  nOHNH | 9.18  1003.95  12  2 | MUT: 10  TER: 10  IRRIT: 10  EF REP: 0 | -20 | 30.35 |
| **61** |  | -11,8 | -10.81 | Y9, D10, E13, I14, K17, A77, P75, G83, P84, L86, P90, P91, Q92, H94, F210  0+ | I107, A110, D111, I114, E115, F132, L135, F136, L137, W182, V186  0+ | LogP  MW  nON  nOHNH | 9.08  988.94  11  3 | MUT: 10  TER: 10  IRRIT: 10  EF REP: 10 | -10 | 50.81 |
| **62** |  | -10.74 | -9.98 | E13, I14, K17, H20, Y21, D64, A67, R68, S70, P75, A81, G83, L86, P91, P90, H94 D211  0+ | I107, D111, L129, F132, M133, F136, V139, N140, G179, W182, M183, V186  0+ | LogP  MW  nON  nOHNH | 9.13  1061.99  14  2 | MUT: 10  TER: 10  IRRIT: 10  EF REP: 0 | -45 | 4.98 |
| **63** |  | -8.52 | -9.4 | K17, S70, Q73, P75, P91, H94, L95, L209, F210  0+ | F146, T149 W150, F153, I157, F168, L 176, I181, A184, T220, L221, V225, P226, I229  0+ | LogP  MW  nON  nOHNH | 8.91  1047.96  14  3 | MUT: 10  TER: 10  IRRIT: 10  EF REP: 0 | -30 | 19.4 |
| **64** |  | -9.94 | -11.33 | H20, Y21, Q66, A67, R68, R98, Q99, D103, R106, Y202, G203, M206, R207, P208, L209, F210  0+ | F74, I107, A110, D111, I114, E115, N118, L129, L135, F136, L137, V139, N140, W182  0+ | LogP  MW  nON  nOHNH | 9.63  1079.06  11  2 | MUT: 10  TER: 10  IRRIT: 10  EF REP: 10 | -15 | 46.33 |
| **65** |  | -10,73 | -11.12 | D111, F112, M115, L121, T125, A126, R127, G128, R129, F130, T132, Q133, F153, M157, W176  40+ | F146, T149, W150, K171, H172, R175, L176, I181, A185, T220, L221, V225, P226, I229  0+ | LogP  MW  nON  nOHNH | 9.08  988.94  11  3 | MUT: 10  TER: 10  IRRIT: 10  EF REP: 10 | -5 | 56.12 |
| **66** |  | -12.81 | -11.49 | D10, E13, I14, K17, P78, G83, P84, L86, P90, P91, Q92, H94, L95, R207, L209, F210  0+ | L81, V82, I85, L96, Y97, I99, N100, F147, H173, S177, G179, W182  0+ | LogP  MW  nON  nOHNH | 9.19  1017.94  13  2 | MUT: 10  TER: 10  IRRIT: 10  EF REP: 0 | -40 | 11.49 |
| **67** |  | -10.18 | -9.84 | L119, H120, L121, T122, T125, R127, G128, R129, F130, A131, T132, Q133, F153, M157, W176  10+ | F146, W150, L176, L150, I181, A184, S187, A188, V191, P192, L217, L221, V225  0+ | LogP  MW  nON  nOHNH | 9.13  1017.94  13  3 | MUT: 10  TER: 10  IRRIT: 10  EF REP: 0 | -25 | 24.84 |
| **68** |  | -11.24 | -10.66 | F112, H120, L121, F124, T125, R127, G128, R129, F130, T132, Q133, Q134, F153, M157  30+ | T149, F146, W150, F153, I153, I157, F168, H172, L180, I181, A184, A188, W216, T220, L221, V225, I 229  0+ | LogP  MW  nON  nOHNH | 9.54  1110.08  13  2 | MUT: 10  TER: 10  IRRIT: 10  EF REP: 0 | -30 | 20.66 |
| **69** |  | -11.23 | -10.38 | K17, H20, S24, E29, Q66, A67, T69, H94, L209, F210, D211  0+ | L106, I107, A110, D111, I114, N118, L129, F132, F136, L137, V139, N140  0+ | LogP  MW  nON  nOHNH | 9.11  1002.97  12  3 | MUT: 0  TER: 10  IRRIT: 0  EF REP: 0 | -40 | -10 |
| **70** |  | -10.2 | -10.41 | M115, H120, L121, T122, T125, G128, R129, F130, A131, T132, Q134, L137, F153  40+ | F70, F74, I107, D111, L113, I114, N118, Y124, L129 D125, F132, M133, F136, L137  0+ | LogP  MW  nON  nOHNH | 9.19  1017.00  12  2 | MUT: 0  TER: 10  IRRIT: 0  EF REP: 0 | -40 | -10 |
| **71** |  | -10.51 | -10.6 | E13, K17, H20, Y21, S24, G83, P84, P91, H94, L95, R207, L209  0+ | L81, V82, I85, M91, L96, Y97, I99, N100, L106, F147, H 73, S177, W182  0+ | LogP  MW  nON  nOHNH | 9.49  1079.07  12  3 | MUT: 0  TER: 10  IRRIT: 0  EF REP: 0 | -30 | 0.6 |
| **72** |  | -10.51 | -11.38 | H120, L121, T122, F124, T125, R127, G128, R129, F130, F153, M157, W176  40+ | L69, C318, L319, L322, I323, S325, F326, L327, R332, L335, R336  0+ | LogP  MW  nON  nOHNH | 9.40  1123.08  14  3 | MUT: 0  TER: 10  IRRIT: 0  EF REP: 0 | -25 | 6.38 |
| **73** |  | -9,76 | -10.06 | G41, A42, A34, H120, T122, P123, F124, T125, A126, R127, G128, R129, F153, W176  30+ | N100, I107, D111, E115, L129, F132, M133, F136, L137 V139, N140, W182, S177  0+ | LogP  MW  nON  nOHNH | 8.91  1034.96  13  4 | MUT: 10  TER: 10  IRRIT: 10  EF REP: 10 | -15 | 45.06 |
| **74** |  | -10.98 | -10.01 | D111, F112, M115, L119, H120, T125, A126, R127, G128, R129, F130, T132, Q133, Q134, F153, M157, W176  40+ | N100, I107, D111, E115, L129, F132, M133, F136, L137, V139  0+ | LogP  MW  nON  nOHNH | 9.16  1018.97  12  3 | MUT: 10  TER: 10  IRRIT: 10  EF REP 10 | -15 | 45.01 |
| **75** |  | -11.19 | -10.73 | M115, L119, H120, L121, F124, T125, R127, G128, R129, F130, F153, W176  40+ | F269, V270, L273, P274, V277, F278, V281, F298, A301, H302, T305, G306, I308, V309, A312  8.3+ | LogP  MW  nON  nOHNH | 9.30  1002.97  11  2 | MUT:10  TER: 10  IRRIT: 10  EF REP: 10 | -20 | 49.03 |
| **76** |  | -11.15 | -10.79 | E13, K17, H20, G83, P91, H94, L95, R207, L209, F210, D211  0+ | L81, V82, I85, N100, V108, L135, N140, S177, G179, W182, M183, V186  0+ | LogP  MW  nON  nOHNH | 9.01  989.93  12  3 | MUT: 10  TER: 10  IRRIT: 10  EF REP: 0 | -5 | 45.79 |
| **77** |  | -10.75 | -11.8 | M16, K17, H20, Y21, S24, E29, R68, A82, G83, P91, Q92, H94, L95, L209, F210, D211  0+ | Y234, L241, V242, R259, L262, V265, V267, F269, V270, I323, F326, L327  0+ | LogP  MW  nON  nOHNH | 9.49  1139.14  12  2 | MUT: 0  TER: 10  IRRIT: 0  EF REP: 0 | -30 | 1.8 |
| **78** |  | -13.7 | -13.11 | M115, L119, H120, L121, T125, G128, R129, F130, T132, Q133, Q134, F153, M157, W17  40+ | L81, N100, V103, I107, F136, N140, F147, L176, S177, C178, G179, I181, W182  0+ | LogP  MW  nON  nOHNH | 9.24  1003.95  12  2 | MUT: 10  TER: 10  IRRIT: 10  EF REP: 0 | -25 | 28.11 |
| **79** |  | -9.7 | -10.85 | L119, H120, L121, T122, T125, G128, R129, F130, T132, F153, M157, R164  30+ | F227, G231, Y234, L241, R259, L262, A263, V265, L266, F269, L273, P274  0+ | LogP  MW  nON  nOHNH | 9.05  1018.97  13  4 | MUT: 0  TER: 0  IRRIT: 10  EF REP: 0 | -20 | 10.85 |
| **80** | NOT PROCESSED | | | | | | | | | |
| **81** |  | -9.98 | -9.51 | K17, Y21, D64, A67, P75, A77, P84, P88, P91, H94, L95, F210, D211  0+ | V103, L106, I107, D111, I114, E115, L129, F132, M133, F136, L137, N140, W182, V186  0+ | LogP  MW  nON  nOHNH | 9.66  1150.14  13  2 | MUT: 0  TER: 10  IRRIT: 0  EF REP: 10 | -30 | 9.51 |
| **82** |  | -12.3 | -10.9 | D10, E13, I14, K17, Q73, P75, P78, G83, L86, P90, P91, H94, F210  0+ | F146, W150, L176, L180, I181, A184, S187, A188, V191, L217, T220, L221, V225, P226, I229  0+ | LogP  MW  nON  nOHNH | 9.34  1023.38  11  3 | MUT: 10  TER: 10  IRRIT: 10  EF REP: 10 | -5 | 55.9 |
| **83** |  | -9.49 | -8.73 | E13, M16, K17, H20 Y21, S24, E29, D31, A32, G33, A67, R68, T69, H94, D171, F210  0+ | L81, R88, E89, M91, I99, N100, V103, I107, F136, V139, N140, W182  0+ | LogP  MW  nON  nOHNH | 9.43  1154.16  13  4 | MUT: 0  TER: 10  IRRIT: 10  EF REP: 0 | -20 | 18.73 |
| **84** | NOT PROCESSED | | | | | | | | | |
| **85** |  | -8.99 | -10.85 | M115, L119, H120, L121, T122, T125, R129, F130, F153, R164  20+ | F227, A228, G231, Y234, V238, L241, V242, R259, L262, L266, F269, V270, L273  0+ | LogP  MW  nON  nOHNH | 9.03  1048.99  13  3 | MUT: 10  TER: 10  IRRIT: 10  EF REP: 10 | -25 | 35.85 |
| **86** |  | -9.07 | -9.91 | D64, P65, Q66, A67, S70, L72, Q73, T74, P75, A76, P91, Q92, H94, L95, L209, F210  0+ | D111, I114, E115, N118, Y124, V128, L129, F132, M133, F136, L137  0+ | LogP  MW  nON  nOHNH | 9.47  1184.18  14  4 | MUT: 0  TER: 10  IRRIT: 0  EF REP: 10 | -25 | 14.91 |
| **87** |  | -10.2 | 11.82 | D10, E13, I14, K17, L86, P90, P91, H94  0+ | Q53, Q54, G58, L59, S62, F206, F208, F223, E275, N276, F278, I279, P303, N310  58+ | LogP  MW  nON  nOHNH | 6.37  567.82  7  3 | MUT: 10  TER: 10  IRRIT: 10  EF REP: 0 | -15 | 104.82 |
| **88** |  | -10.1 | -10.28 | D10, E13, I14, K17, L86, P90, P91, H94, F210  0+ | Q53, G58, L59, S62, F206, F208, F223, E275, N276, F278, I279, P303, N310  50+ | LogP  MW  nON  nOHNH | 5.72  533.38  7  3 | MUT: 10  TER: 10  IRRIT: 10  EF REP: 10 | -15 | 105.28 |
| **89** |  | -8.84 | 10.32 | D10, I14, K17, L86, P90, P91, H94, F210  0+ | L108, S112, E115, L137, M141, F206, F208, W272, I279, A313, F314, S317  8+ | LogP  MW  nON  nOHNH | 5.20  505.37  6  3 | MUT: 10  TER: 10  IRRIT: 10  EF REP: 10 | 0 | 78.32 |
| **90** |  | -9.28 | -9.58 | F112, M115, L119, A126, G128, F130, A131, T132, Q133, Q134, F153, M157, W176  30+ | Q53, Q54, G58, L59, S62, L119, C205, C207, F208, E275, F278, I279, H282, R286, P303, N310  83+ | LogP  MW  nON  nOHNH | 6.41  601.38  7  3 | MUT: 10  TER: 10  IRRIT: 10  EF REP: 10 | -20 | 132.58 |
| **91** |  | -8.49 | -10.93 | F112, G128, R129, F130, A131, T132, Q133, Q134, F153  40+ | H52, Q53, Q54, G58, L119, A204, C205, F206, C207, F208, E275, F278, I 279, R286, P303, H307, N310  91+ | LogP  MW  nON  nOHNH | 5.86  551.37  7  3 | MUT: 10  TER: 10  IRRIT: 10  EF REP: 10 | -20 | 141.93 |
| **92** |  | -9.46 | -10.06 | D111, F112, R129, F130, A131, T132, Q133, Q134, L137, A149, F153  30+ | Q53, Q54, G58, L119 C205, F206, E275, F278, I279, R286, P303, H307, N310  75+ | LogP  MW  nON  nOHNH | 5.83  567.82  7  3 | MUT: 10  TER: 10  IRRIT: 10  EF REP: 10 | -15 | 130.06 |
| **93** |  | -9.84 | -11.72 | -9.84 | H52, Q53, Q54, G58, L119, A204, C205, F206, C207, F208, E275, F278, I 279, R286, P303, H307, N310  91+ | LogP  MW  nON  nOHNH | 6.50  612.27  7  3 | MUT: 10  TER: 10  IRRIT: 10  EF REP: 10 | -10 | 152.72 |
| **94** |  | -9.42 | -12.08 | L121, A126, G128, R129, F130, A131, T132, Q133, Q134, F153, M157  40+ | Q53 Q54, G58, L59, S62, L119, Y123, F206, C207, F208, F278, P303, G306, H307, N310  83+ | LogP  MW  nON  nOHNH | 6.50  612.27  7  3 | MUT: 10  TER: 10  IRRIT: 10  EF REP: 10 | -15 | 140.08 |
| **95** |  | -9.75 | -10.08 | Q118, L119, H120, R129, F153, M157  10+ | Q53, A204, C205, C207, F208, E218, E275, N276, F278, I279, N310  41+ | LogP  MW  nON  nOHNH | 6.46  602.27  7  3 | MUT: 10  TER: 10  IRRIT: 5  EF REP: 10 | -25 | 81.08 |
| **96** |  | -9.86 | -11.46 | T7, G8, Y9, D10, E13, I14, K17, A82, G83, L86, S87, P91, H94  0+ | L108, L137, M141, Y142, F208, F223, W272, E275, N276  0+ | LogP  MW  nON  nOHNH | 6.09  529.43  5  2 | MUT: 10  TER: 10  IRRIT: 10  EF REP: 10 | -30 | 41.46 |
| **97** |  | -10,32 | -9.83 | D111, T125, R129, F130, T132, Q133, Q134, F153  10+ | I 230, G231, R259, L262, V265, A263, V265, L266, F269  0+ | LogP  MW  nON  nOHNH | 7.24  691.17  7  3 | MUT: 10  TER: 10  IRRIT: 10  EF REP: 10 | -10 | 59.83 |
| **98** |  | -8.64 | -8.82 | D111, F112, M115, R127, G128, R129, F130, T132, F153  30+ | Y234, R259, L262, A263, V265, L266, F269, L327  0+ | LogP  MW  nON  nOHNH | 6.66  617.37  8  3 | MUT: 10  TER: 10  IRRIT: 10  EF REP: 10 | -25 | 43.82 |
| **99** |  | -9,43 | -10.26 | Y9, E13, I14, K17, L86, P90, P91, H94, F210  0+ | Q53, Q54 G58, L59, S62, F206, F208, F223, E275, N276, F278, I279, P303, N310  58+ | LogP  MW  nON  nOHNH | 5.95  569.36  7  3 | MUT: 10  TER: 10  IRRIT: 10  EF REP: 10 | -10 | 118.26 |
| **100** |  | -9.4 | -10.47 | Y9, D10, N11, R12, E13, I14, K17, L86, P91, N182,  0+ | Q138, M141, Y142, F206, F208, E218, F223, W272, E275, F278, I279  8+ | LogP  MW  nON  nOHNH | 5.43  596.52  8  2 | MUT: 10  T: 10  IRRIT: 10  EF REP: 10 | -20 | 58.47 |
| **101** |  | -8.8 | -9.89 | Y9, D10, E13, I14, K17, L86, P90, P91, H94, F210  0+ | H52, Q53, F206, F208, Q215 E218, V219, F223, E275, F278, I279, H282  33+ | LogP  MW  nON  nOHNH | 6.46  585.81  7  3 | MUT: 10  TER: 10  IRRIT: 10  EF REP: 10 | -10 | 92.89 |
| **102** |  | -10.18 | -10.22 | F112, F124, T125, R127, G128, R129, F130, T132, F153  30+ | Y97, W150, F153, D154, I157, F168, R169, H172, H 73, L176, L180, A184, M183  0+ | LogP  MW  nON  nOHNH | 7.35  639.50  8  3 | MUT: 10  TER: 10  IRRIT: 10  EF REP: 10 | -45 | 25.22 |
| **103** |  | 8.9 | -9.89 | Y9, D10, E13, I14, K17, L86, P90, P91, H94, F210  0+ | Y97, F136, W150, F153, D154, I157, F168, R169, H172, H173, L176, L180 A184 V211  0+ | LogP  MW  nON  nOHNH | 8.17  658.06  6  2 | MUT: 10  TER: 10  IRRIT: 0  EF REP: 10 | -5 | 35.89 |
| **104** |  | -9.38 | -9.5 | F112, L119, H120, T122, T125, F130, A131, T132, Q133, Q134, L137, F153, M157  20+ | L135, F136, Q138, V139, N140, M141, Y142, S143, F146, W182, S185, V186, S187, A188, T189, T220, L221, V225  0+ | LogP  MW  nON  nOHNH | 6.46  585.81  7  3 | MUT: 10  TER: 10  IRRIT: 10  EF REP: 10 | -55 | 14.5 |
| **105** |  | -9.44 | -9.51 | I14, Q15, M16, K17, Y18, I19, L97, R98, F151, G154, C158, L169, Q170, D171, N172, I173, A174, L175, M177  0+ | I107, I114, N118, F132, F136, V139, N140, W182, V186  0+ | LogP  MW  nON  nOHNH | 6.00  573.40  8  3 | MUT: 10  TER: 10  IRRIT: 10  EF REP: 10 | -30 | 39.51 |
| **106** |  | -9.41 | -9.95 | I14, Q15, M16, K17, Y18, I19, L97, R98, F152, G154, C158, L169, Q170, D171, N172, I173, A174, L175, M177  0+ | H52, Q53, L119, Q138, Y142, C205, F206, F208, E275, N276, F278, I279, H282, P303  58+ | LogP  MW  nON  nOHNH | 5.58  577.38  9  3 | MUT: 10  TER: 10  IRRIT: 10  EF REP: 10 | -85 | 42.95 |
| **107** |  | 8.65 | -9.9 | K17, H20, Y21, S24, E29, D31, D64, Q66, A67, R68, S70, Q73, H94, F210, D211  0+ | I107, N118, F132, F136, Q138, V139, N140, F153, W182, V186  0% | LogP  MW  nON  nOHNH | 7.58  623.61  6  2 | MUT: 10  TER: 10  IRRIT: 0  EF REP: 10 | -10 | 49.9 |
| **108** |  | -11.9 | -11.22 | K8, Y9, D10, E13, I14, K17, H20, P78, A81, A82, G83, L86, P90, W195  0+ | F146, W150, F153, D154, H172, L176, L180, I182, A184, L221, P226  0+ | LogP  MW  nON  nOHNH | 6.83  692.61  8  2 | MUT: 10  TER: 10  IRRIT: 10  EF REP: 10 | -25 | 46.22 |
| **109** |  | -9.5 | -10.53 | D111, F112, M115, L119, H120, L121, T125, F130, A131, T132, Q133, Q134, L137, F153  20+ | V103, I107, N118, A119, Y124, L129, F132, M133, F136, N140, V139, W182, V186  8+ | LogP  MW  nON  nOHNH | 9.41  1089.06  14  3 | MUT: 0  TER: 10  IRRIT: 0  EF REP: 0 | -10 | 22.53 |
| **110** |  | -10.84 | -10.37 | L119, H120, L121, T122, T125, A126, G128, R129, F130, A131, T132, Q133, Q134, L137, F153, M157, W176  50+ | F146, W150, F153, H172, L176, I 181, A184, V225, P226, I229, L232  0+ | LogP  MW  nON  nOHNH | 9.77  1081.04  12  1 | MUT: 10  TER: 10  IRRIT: 10  EF REP: 0 | -15 | 35.37 |
| **111** |  | -8.89 | -9.73 | K17, H20, Y21, S24, E29, D31, D64, Q66, A67, R68, S70, Q73, H94, F210, D211  0+ | F146, W150, R175, L176, L180, I181, M183, A184, S187, A188, L217, L221, V225, P226  0+ | LogP  MW  nON  nOHNH | 9.39  1134.10  15  4 | MUT: 0  TER: 10  IRRIT: 0  EF REP: 10 | -15 | 24.74 |
| **112** |  | -9.12 | -11.77 | R6, T74, A77, P78, P84, P88, Q89, P91, Q92, H94, L95, R207, P208, L209  0+ | V103, L106, I107, A110, D111, I114, E115, N118, L129, M133, L135, F136, L137, V139, W182, V186  0+ | LogP  MW  nON  nOHNH | 9.79  1194.20  14  3 | MUT: 0  TER: 10  IRRIT: 0  EF REP: 10 | -15 | 26.77 |
| **113** |  | -10.69 | -9.68 | D111, F124, T125, A126, R127, G128, R129, F130 A131, T132, Q134, F153  40+ | V39, L81, I85, E89, L91, L95, I99, N100, I107, F136, V139, W182  0+ | LogP  MW  nON  nOHNH | 8.80  859.73  11  4 | MUT: 0  TER: 10  IRRIT: 0  EF REP: 10 | -55 | -16-68 |
| **114** |  | -10.19 | -10.28 | R12, E13, I14, Q15, M16, K17, Y18, I19, W30, Q93, H94, T96, L97, G101, Q148, F150, E152, F151, E152, F153, G154, G155, Q156, C158, Q170, D171, I173, A174, L175, W176, M177  10+ | F146, W150, F153, L176, L180, I181, A184, S187, A188, T220, L221, V225  0+ | LogP  MW  nON  nOHNH | 9.47  949.86  11  3 | MUT: 0  TER: 10  IRRIT: 0  EF REP: 10 | -65 | -25.28 |
| **115** |  | -11.8 | -11.01 | K17, P75, A76, P78, G79, A82, G83, P84, P91, Q92, P94, L95, R207, L209, F210, D211  0+ | F146, W150, F153, D154, H172, L176, A184, A188, L221, V225, P226  0+ | LogP  MW  nON  nOHNH | 9.76  1081.04  12  1 | MUT: 10  TER: 10  IRRIT: 10  EF REP: 0 | -20 | 30.01 |
| **116** |  | -10,73 | -10.12 | F112, M115, Q118, L119, L121, A126, R127, G128, F130, A131, T132, Q133, Q134, F153, M157, W176  40+ | W150, F153, I157, R161, F168, H172, L176, L180  0+ | LogP  MW  nON  nOHNH | 7.16  858.95  12  3 | MUT: 10  TER: 10  IRRIT: 10  EF REP: 10 | -5 | 55.12 |


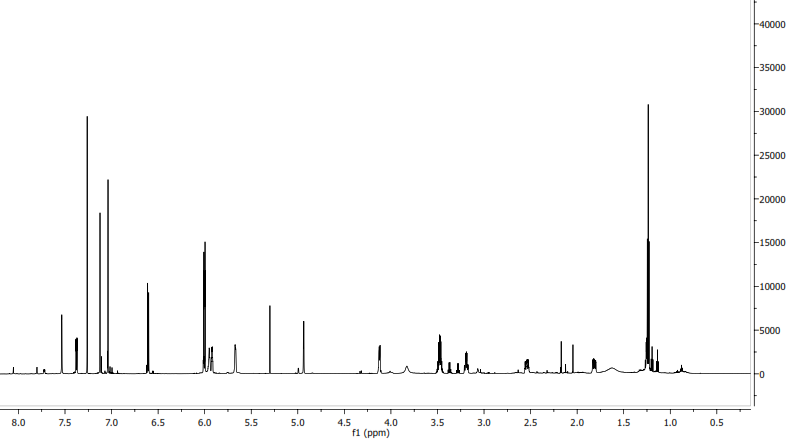


**Figure S1.** ^1^H NMR spectra of compound **6**.

**Figure S2.** ^13^C NMR spectra of compound **6**.


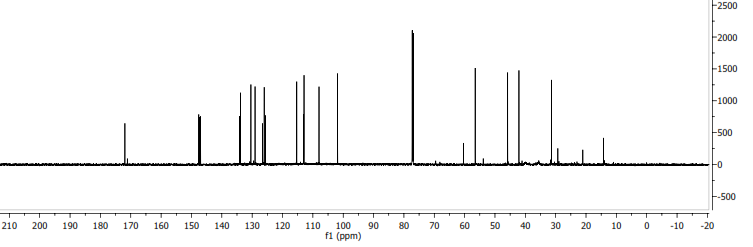


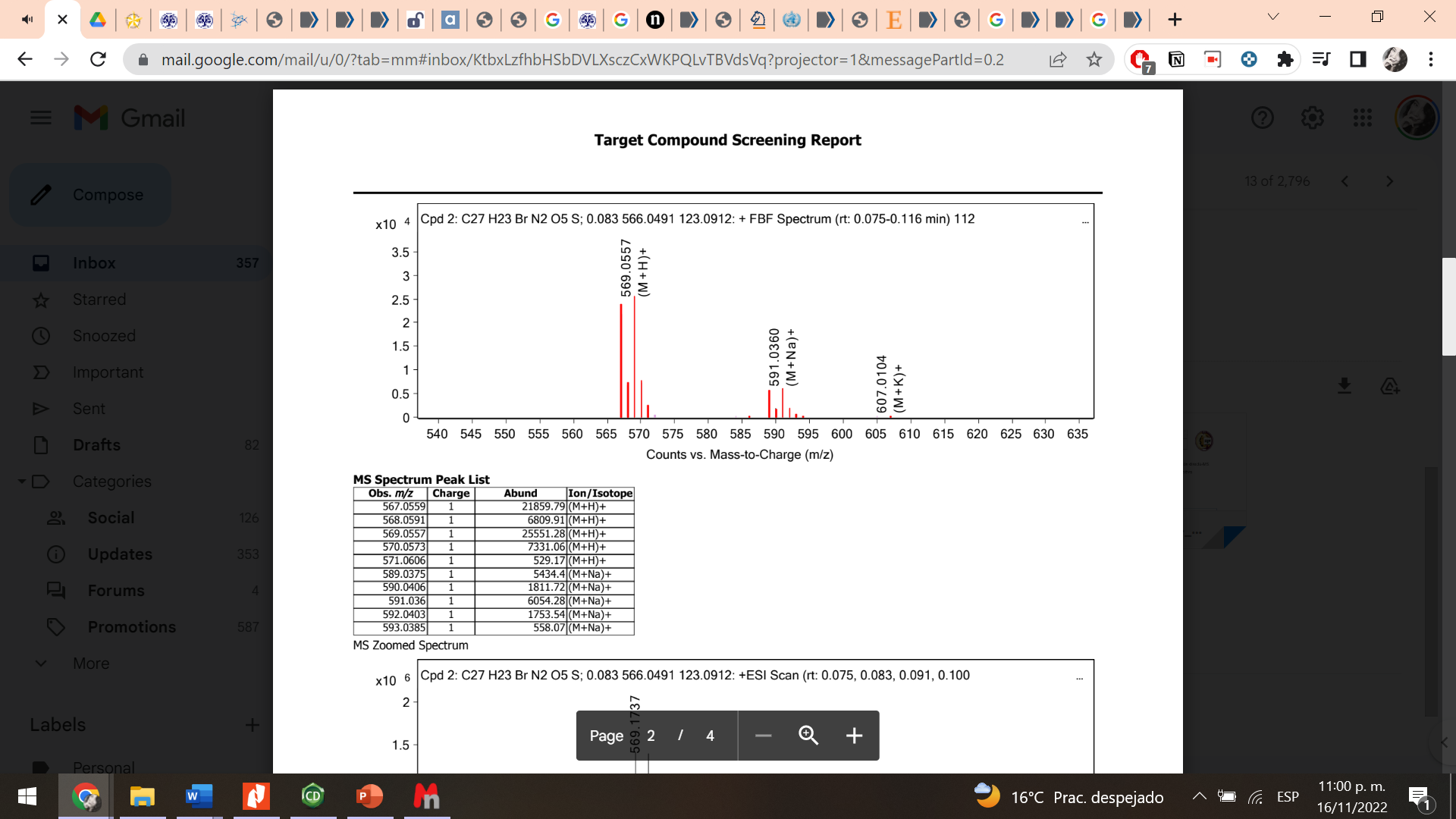

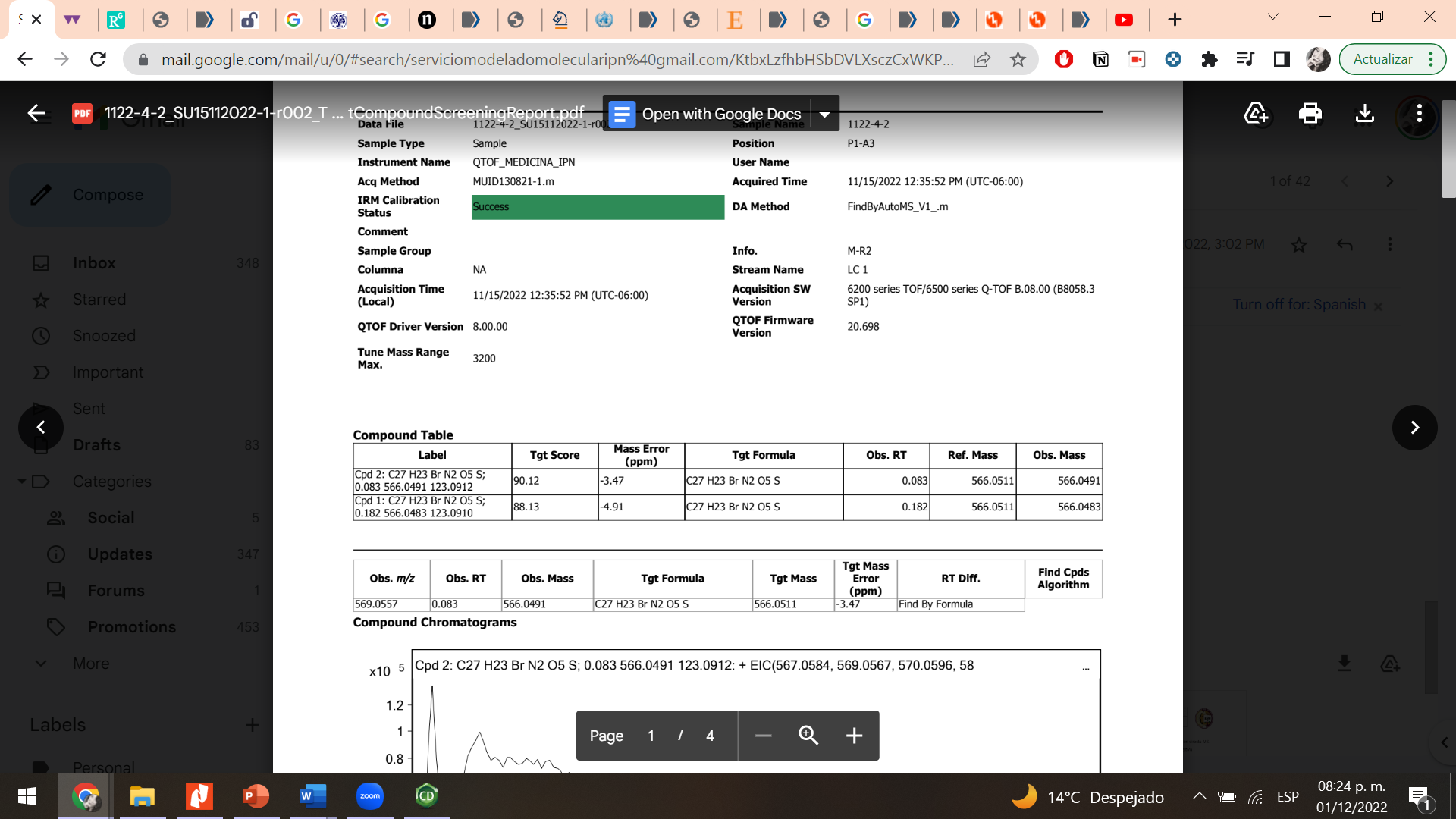


**Figure S3.** HPLC-MS spectra and isotopic distribution of compound **6**.

|  | 6 | |
| --- | --- | --- |
|  | ^1^H | ^13^C |
| 1 | 5.99 – 6.03 (d, J= 10.7 Hz) | 113.05 |
| 2 | 5.99 – 6.03 (d, J= 10.7 Hz) | 130.41 |
| 3 | 1.82 (dd, J = 17.3, 8.5 Hz)  2.54 (dd, J = 15.0, 10.9 Hz) | 31.42 |
| 3a | 3.19 (dd, J=9.5, 3.3 Hz) | 42.10 |
| 4 | 4.94 (d, J= 3.1 Hz) | 56.52 |
| NH |  | - |
| 6 | 7.12 (d, J= 12.2 Hz) | 115.30 |
| 7 | 7.38 (dd, J= 8.3, 2.0) | 129.05 |
| 8 | - | 134.13 |
| 9 | 7.54 (d, J=1.3 Hz) | 129.47 |
| 9a | - | 133.86 |
| 9b | 4.12 (d, J=7.3 Hz) | 45.80 |
| 2’ | 5.92 – 5.95 (d, J= 13.6 Hz) | 101.82 |
| 3’a | - | 147.47 |
| 4’ | 5.30 (s) | 107.97 |
| 5’ | - | 146.00 |
| 6’ | - | 125.55 |
| 7’ | 5.67 (s) | 112.91 |
| 7’a | - | 148.34 |
| 1’’ | - | 133.86 |
| 2’’ | 6.61 (d, J= 8.4 Hz) | 126.04 |
| 3’’ | 7.04 (d, J=4.6 Hz) | 126.53 |
| 4’’ | - | 125.55 |
| 5’’ | 7.04 (d, J=4.6 Hz) | 126.53 |
| 6’’ | 6.61 (d, J= 8.4 Hz) | 126.04 |
| CH_3_ | 1.25 | 21.04 |

**Table S2.** ^1^H and ^13^C chemical shifts form compound **6**.


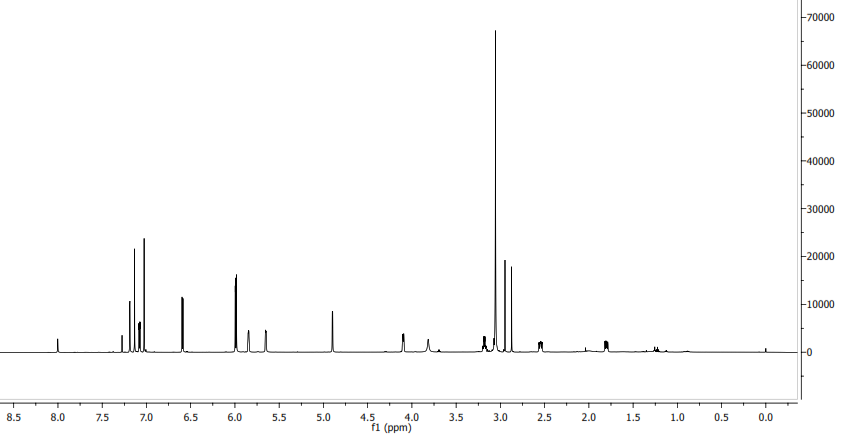

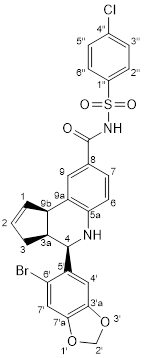


**Figure S4.** ^1^H NMR spectra of compound **37**.

**Figure S5.** ^13^C NMR spectra of compound **37**.


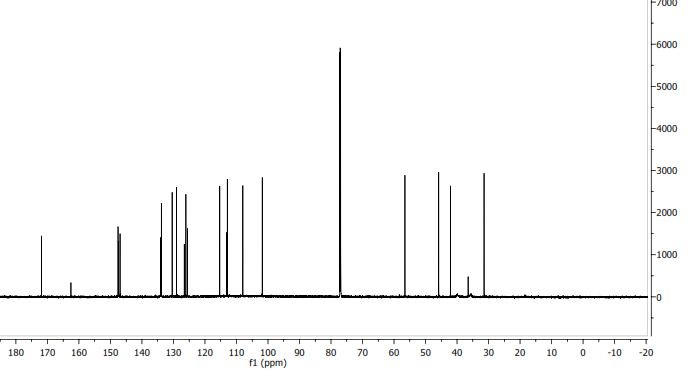

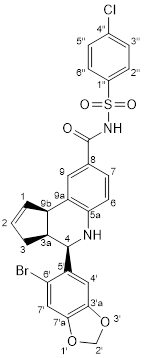


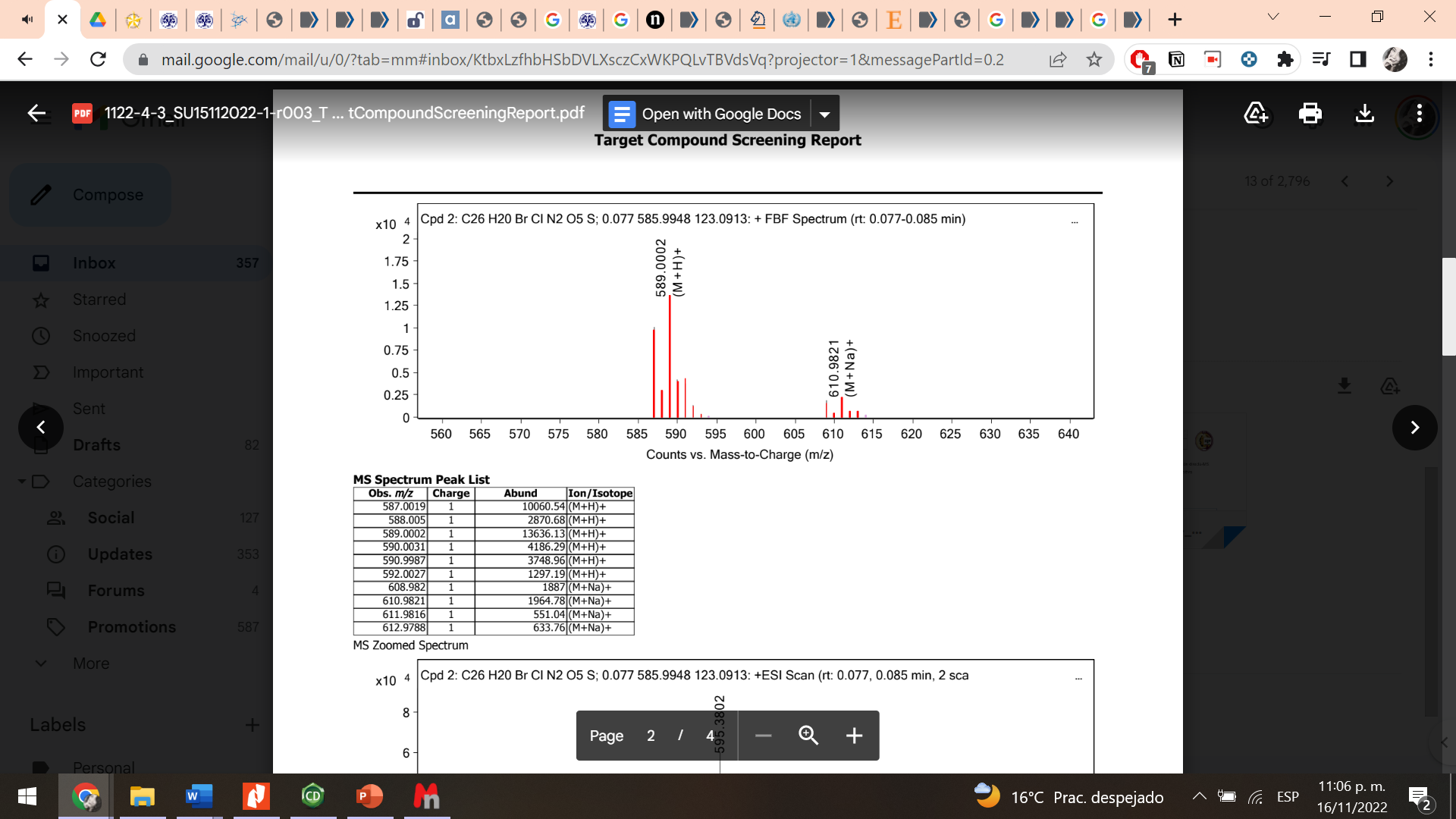


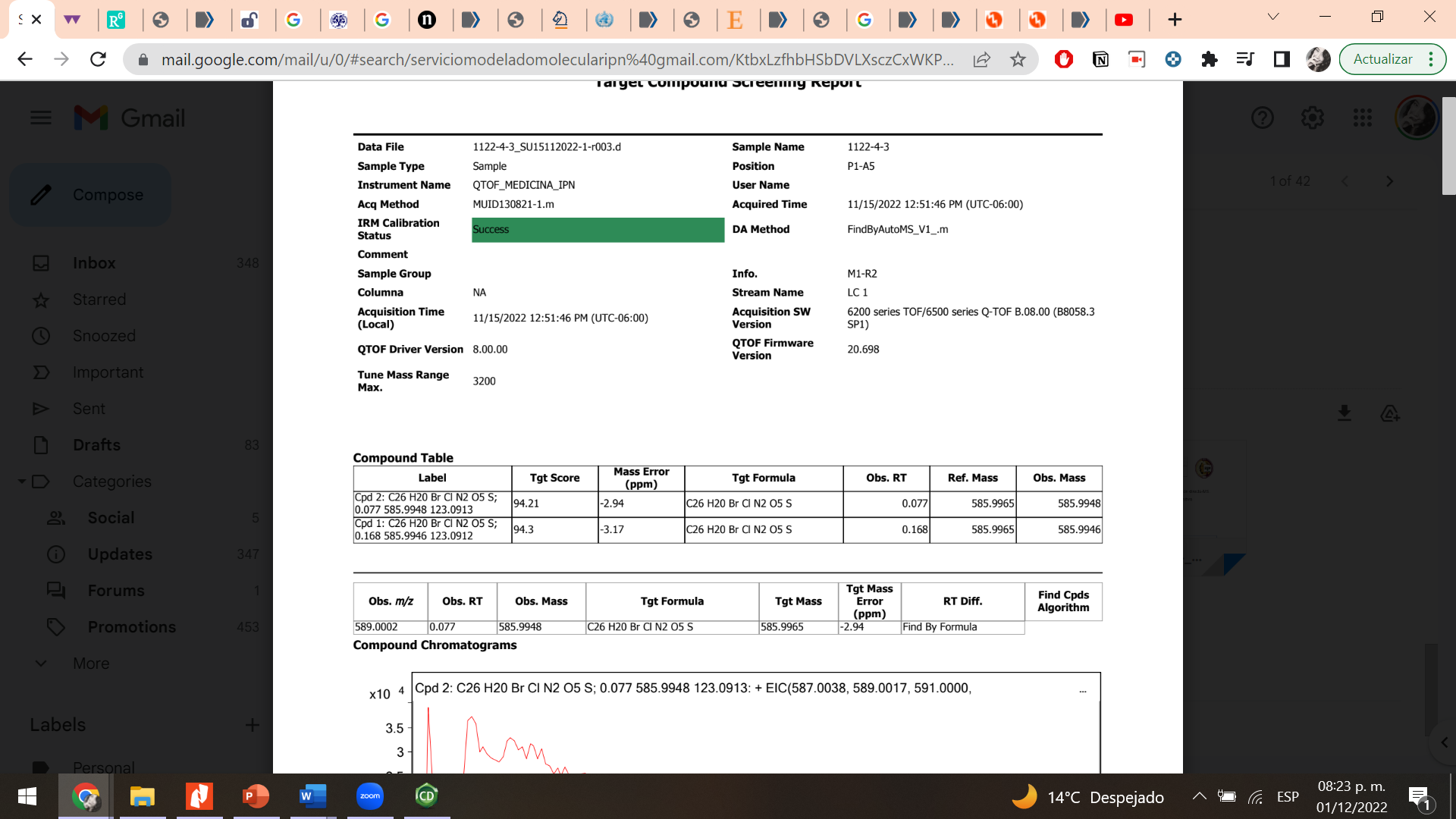


**Figure S6.** HPLC-MS (ESI) spectra and isotopic distribution of compound **37**.

|  | 37 | |
| --- | --- | --- |
|  | ^1^H | ^13^C |
| 1 | 5.99 (d, J= 9.8 Hz) | 113.06 |
| 2 | 5.99 (d, J= 9.8 Hz) | 130.42 |
| 3 | 1.80 (dd, J = 15.6, 8.9 Hz  2.54 (dd, J = 14.1, 11.8 Hz) | 31.43 |
| 3a | 3.18 (dd, J = 9.0, 3.3 Hz | 42.10 |
| 4 | 4.90 (d, J=3.3 Hz) | 56.53 |
| NH |  |  |
| 6 | 7.13 | 115.29 |
| 7 | 7.08 (dd, J= 8.3, 1.9) | 129.05 |
| 8 | - | 134.13 |
| 9 | 7.19 | 129.05 |
| 9a | - | 133.86 |
| 9b | 4.10 (d, J=8,6 Hz) | 45.80 |
| 2’ | 5.99 (d, J= 9.8 Hz) | 101.82 |
| 3’a | - | 147.38 |
| 4’ | 5.65 (s) | 107.97 |
| 5’ | - | 146.94 |
| 6’ | - | 125.57 |
| 7’ | 5.85 (s) | 112.92 |
| 7’a | - | 147.58 |
| 1’’ | - | 133.86 |
| 2’’ | 6.59 (d, J= 8.2 Hz) | 126.05 |
| 3’’ | 7.07 (d, J=1.9 Hz) | 126.57 |
| 4’’ | - | 125.57 |
| 5’’ | 7.07 (d, J=1.9 Hz) | 126.57 |
| 6’’ | 6.59 (d, J= 8.2 Hz) | 126.05 |

**Table S3.** ^1^H and ^13^C chemical shifts form compound **37**.
